# Generative artificial intelligence in drug discovery: basic framework, recent advances, challenges, and opportunities

**DOI:** 10.3389/fphar.2024.1331062

**Published:** 2024-02-07

**Authors:** Amit Gangwal, Azim Ansari, Iqrar Ahmad, Abul Kalam Azad, Vinoth Kumarasamy, Vetriselvan Subramaniyan, Ling Shing Wong

**Affiliations:** ^1^ Department of Natural Product Chemistry, Shri Vile Parle Kelavani Mandal’s Institute of Pharmacy, Dhule, Maharashtra, India; ^2^ Computer Aided Drug Design Center Shri Vile Parle Kelavani Mandal’s Institute of Pharmacy, Dhule, Maharashtra, India; ^3^ Department of Pharmaceutical Chemistry, Prof. Ravindra Nikam College of Pharmacy, Dhule, India; ^4^ Faculty of Pharmacy, University College of MAIWP International, Batu Caves, Malaysia; ^5^ Department of Parasitology and Medical Entomology, Faculty of Medicine, Universiti Kebangsaan Malaysia, Cheras, Malaysia; ^6^ Pharmacology Unit, Jeffrey Cheah School of Medicine and Health Sciences, Monash University Malaysia, Selangor, Malaysia; ^7^ School of Bioengineering and Biosciences, Lovely Professional University, Phagwara, Punjab, India; ^8^ Faculty of Health and Life Sciences, INTI International University, Nilai, Malaysia

**Keywords:** ChatGPT, *de novo* drug design, deep generative models, AlphaFold, variational autoencoders, generative adversarial network, large language models, chemical language models

## Abstract

There are two main ways to discover or design small drug molecules. The first involves fine-tuning existing molecules or commercially successful drugs through quantitative structure-activity relationships and virtual screening. The second approach involves generating new molecules through *de novo* drug design or inverse quantitative structure-activity relationship. Both methods aim to get a drug molecule with the best pharmacokinetic and pharmacodynamic profiles. However, bringing a new drug to market is an expensive and time-consuming endeavor, with the average cost being estimated at around $2.5 billion. One of the biggest challenges is screening the vast number of potential drug candidates to find one that is both safe and effective. The development of artificial intelligence in recent years has been phenomenal, ushering in a revolution in many fields. The field of pharmaceutical sciences has also significantly benefited from multiple applications of artificial intelligence, especially drug discovery projects. Artificial intelligence models are finding use in molecular property prediction, molecule generation, virtual screening, synthesis planning, repurposing, among others. Lately, generative artificial intelligence has gained popularity across domains for its ability to generate entirely new data, such as images, sentences, audios, videos, novel chemical molecules, etc. Generative artificial intelligence has also delivered promising results in drug discovery and development. This review article delves into the fundamentals and framework of various generative artificial intelligence models in the context of drug discovery via *de novo* drug design approach. Various basic and advanced models have been discussed, along with their recent applications. The review also explores recent examples and advances in the generative artificial intelligence approach, as well as the challenges and ongoing efforts to fully harness the potential of generative artificial intelligence in generating novel drug molecules in a faster and more affordable manner. Some clinical-level assets generated form generative artificial intelligence have also been discussed in this review to show the ever-increasing application of artificial intelligence in drug discovery through commercial partnerships.

## 1 Introduction

New medication development is an expensive and time-consuming process. As a result of concerns about risk and acceptability, the success rate is low. The pharmaceutical sector is seeing a decline in productivity as the cost of developing and bringing a new medicine to market continues to rise (Paul et al., 2010; DiMasi et al., 2016). New processing hardware and novel and improved deep learning (DL) algorithms have increased the success of artificial intelligence (AI) in numerous areas, including computer vision, autonomous vehicles, robotics, and others (von Ungern-Sternberg, 2017; Coeckelbergh, 2020; Thierer et al., 2017; Collins and Moons, 2019). In recent years, DeepMind has developed two Go-playing AI algorithms (AlphaGo and AlphaGo Zero) that can now compete and even win against the best human players (Singh et al., 2017; Chao et al., 2018). Additionally, DeepMind has developed AlphaFold to address the protein-folding challenge, which has been regarded for a long time as a challenging biological problem. AlphaFold 2 performed better than any other technique in the 14th Critical Assessment of Protein Structure Prediction (CASP) (Service, 2020). These advances show the potential of AI to revolutionize basic research by drastically speeding up the pace of research. Motivated by these promising outcomes, medicinal researchers are exploring and utilizing how AI may be used in the pharmaceutical industry. Many pharmaceutical firms have partnered with AI startups and academic institutions or launched in-house AI initiatives. Various biomedical and drug discovery research branches have recently incorporated AI approaches, such as developing deep neural networks using transcriptional data to predict biologically active molecules (Aliper et al., 2016), and generating new small molecule therapeutic leads (Mamoshina et al., 2016). Ongoing studies reveal that AI is being used to revolutionize every step of the drug discovery process, from choosing targets to generating ligands, planning synthesis, selecting trial participants to repurpose existing drugs, analyzing cellular images, and making predictions about molecules’ physicochemical and biological properties.

Numerous criteria, including ADMET (absorption, distribution, metabolism, excretion, and toxicity) and synthesis feasibility, must be satisfied by each potential drug candidate. Therefore, predicting molecular characteristics with high precision is crucial in the drug development process. To save money on R&D, computational studies might screen and enhance projected molecular features before costly animal and clinical testing. For instance, predicting the inhibition of human cytochrome 450 (CYP450) using a multitask deep autoencoder was described by Li et al. (Li et al., 2018a), paving the way for minimizing adverse effects and drug interactions. To predict the aqueous solubility and logP of small compounds, Tang and coworkers employed a deep self-attention message-passing graph neural network (SAMPN) (Tang et al., 2020). In addition, their SAMPN model identified the primary structural characteristic for a target attribute, which shed interpretable light on the DL “black box” issue. Leads may also be found using neural networks trained to predict chemical properties. For instance, Stokes et al. (Stokes et al., 2020), used a deep neural network based on molecular graphs to predict antibiotic activity in compounds structurally distinct from known antibiotics and then validated it in experiments.

However, these methods have significant drawbacks in common, including the need for colossal training datasets and well-trained neural networks to learn the abstract representation of molecules, molecular grammar, molecular properties, and chemical aspects. Another point is that this prediction-based approach cannot generate new compounds if the basic or essential molecules are missing from the input chemical library. Here lies the role of generative AI (GAI). GAI is a type of AI that uses DL to generate novel content. GAI tools understand the patterns and intricacy of their training data and then generate new data (either similar or improved version per the user’s expectation). New data can be text, images, audio, video, chemical molecules, etc., based on patterns they have learned from training data. GAI is being used to generate content in response to user-given prompts. GAI is different from traditional AI systems, which are trained to examine data and make predictions based on pattern recognition.

One of the most appealing features of GAI-driven drug development is the ability of GAI models to produce completely novel compounds. Generating unique or novel molecular structures with desired features is known as *de novo* molecular generation. As a result, GAI models often include neural networks capable of predicting a given attribute. For example, in a representative work, Popova et al. (Popova et al., 2018), presented ReLeaSE, which associates a neural network (to predict molecular property) with a deep generative neural network (to design novel molecules with the necessary physicochemical and biological characteristics). However, the GAI model developed by Popova et al. was not verified by actual experiments. Subsequently, Merk et al. (Merk et al., 2018a), trained a GAI model on natural products to produce *de novo* ligands, and the resulting molecules were empirically verified as new retinoid X receptor (RXR) modulators. In another scientific breakthrough, to combat fibrosis, Hongkong/New York-based Insilico Medicine developed a GAI model GENTRL (Generative Tensorial Reinforcement Learning) to identify novel kinase DDR1 inhibitors. They performed biological tests to confirm the efficacy of the GENTRL-generated compounds. Their research stood out because they went from insilico to a successful preclinical phase in only 21 days, an achievement that had never been accomplished before (Zhavoronkov et al., 2019). Korshunova et al. recently employed RL, combining transfer learning with the policy gradient algorithm, experience replay, and real-time rewards to generate new EGFR inhibitors that have also been empirically verified (Korshunova et al., 2021).

These amazing use cases of GAI models provide hope for improved methods of producing novel therapeutic molecules from scratch. These examples demonstrate the potential effectiveness of a GAI-driven approach to the drug development process. It should be noted, however, that there is currently no one-size-fits-all approach to AI-driven drug development. For instance, the hyperparameters, GAI frameworks, and model training for any AI-driven drug discovery project may need to be fine-tuned extensively. The biggest obstacles to AI-driven drug development are the immense rounds of trial and error, the absence of a generic/universal mathematical system, and the lack of adequate data.

Furthermore, given the complexity of biological systems, it is unlikely that any of the aforementioned GAI models would continue to be effective if the aim were changed. For instance, there may not be enough data for the predictor and generator to produce drug-like compounds if the target is altered from A to A1 or B. The quantity and quality of the training sets determine the performance of the predictor or classifier or generator. While GAI frameworks and uses have been widely discussed among the core DL fraternity (Salakhutdinov, 2015), their full potential is yet to be tapped (for precise applications/successful use cases) in medicinal chemistry research (Lavecchia, 2019). Even though the aforementioned chain of events—from AI model training to molecular property prediction to the generation of novel drug-like molecules to web lab synthesis and biological validation—remains relatively rare in the scientific literature, GAI-driven drug discovery is emerging as an exciting tool for efficient *de novo* molecule generation. In this review, we introduce various GAI frameworks, explore their uses in drug development, highlight recent successes and challenges, and detail ongoing efforts to address these issues to generate customized needles rather than finding the ones in a haystack. In the Recent Advances section, we have shared comprehensive data about a few under investigation (clinical trial stage) molecules that emanated from generative AI.

## 2 Generative AI

AI models (including machine learning-ML and DL) may, after analyzing patterns/insights in the input data given to them, categorize data (classification), predict numerical output (regression), group data (clustering), and generate either completely new data or data similar to input data. Most of these tasks fall under the purview of either computer vision (which employs the CNN, that is, convolutional neural network type of DL model for analyzing imageries like medical images, other images, and molecular structures in the form of molecular graphs) or natural language processing-NLP (which employs the recurrent neural network-RNN type of DL model for analyzing sequential data like sentences or chemical structures in the form of strings).

**FIGURE 1:**
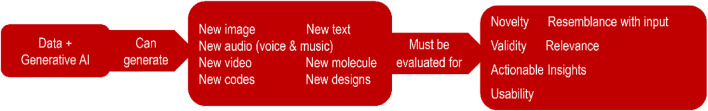
A simple flowchart of the generative AI model’ working.

The capacity of GAI models to generate/design new sets of data (depending on the input data), such as photos, audios, phrases, films, chemical compounds, etc., has recently attracted much interest, mainly because of the success of large language model (LLM). Depending on the GAI model used and the specifics of the work at hand, the results might be exact copies of the inputs or improved versions of inputs (Figure 1).

Since its release in November 2021, OpenAI’s ChatGPT (Chat Generative Pre-trained Transformer) has been one of the most talked-about examples of proprietary GAI. ChatGPT is driven by the LLM (GPT-4) and designed and trained to generate natural-sounding text. Similarly to other LLM-based GAI, it has been trained on large datasets of text and code and can generate creative content, translate languages, generate new text, text similar to the text it was trained on, and answer user-provided prompts in an informative way. ChatGPT, which models human conversation, is the most advanced language simulator accessible today. Its state-of-the-art GAI technology is a transformer architecture learned using large online text datasets. DALL-E 2 (another invention from OpenAI falling in the domain of GAI that allows users to generate new pictures using text-to-graphics prompts) and Midjourney (an AI tool that converts words to images and makes images depending on user prompts) are two other prominent examples of proprietary GAI products (Editorials, 2023; Patel et al., 2023; Shen et al., 2023; Stokel-Walker and Van Noorden, 2023).

As mentioned earlier, discovering and developing new medications has been lengthy and expensive, but the industry has dramatically changed with the introduction of GAI. The GAI method can transform the sector entirely by automating many of the tedious and labor-intensive activities associated with drug development. The use of GAI models for developing therapeutic candidates based on existing biological and chemical information has recently been detailed. For example, NVIDIA’s BioNeMo, Insilico Medicine’s Pharma. AI, etc., are a few examples of GAI tools for the pharma sector (for drug development). NVIDIA’s BioNeMo is a cloud-based GAI platform for the pharmaceutical industry. It is a supercomputing framework for training and deploying massive biomolecular language models. Researchers can rapidly adapt and release scalable generative and predictive biomolecular AI models built using NVIDIA cloud APIs. BioNeMo also allows the implementation of GAI in the generation of protein and biomolecule structure and function, which speeds up the development of novel molecules in the quest for drug discovery. The Pharma. AI platform has a total of three function-specific platforms: PandaOmics, Chemistry42, and inClinico. Through a patented network analysis system called iPanda that deduces pathway activation or inhibition, uncovering linkages between apparently distinct genes basis dysregulated biochemical processes, PandaOmics is developed to allow multi-omics identification of new targets. PandaOmics generates a ranked list of prospective biological targets for a specific illness or subtype of disease and then selects those target theories basis their originality, approachability by small compounds, biologics, and safety. This process yields a potential target score. The company claims that users may discover new lead-like compounds using Chemistry42, the second function-specific platform, which is an automatic, *de novo* drug design and scalable tool. This discovery process can take as little as a week. Chemistry42 employs GAI to generate novel small drug-like molecules, fine-tuned for exact features by drawing on many chemicals and molecular fragments. Chemistry42 establishes standards for innovative compounds’ attributes, including molecular shape, chemical complexity, synthetic accessibility, metabolic stability, etc. A newly generated molecule is first annotated with its characteristics, including physicochemical parameters, binding scores, and drug-likeness traits, before being compared to other compounds in private libraries and vendor catalogues to determine its uniqueness. inClinico is a platform for a data-driven multimodal forecasts of clinical trials’ probability of success (PoS). It uses enormous amounts of data about the targets, ailments, clinical trials, and even researchers involved in the studies.

ChatGPT necessitates this much elaboration since this groundbreaking LLM will shape and reform how existing GAI-guided initiatives go towards *de novo* drug design (Liu et al., 2021; Chakraborty et al., 2023; Chen et al., 2023). There has been one recent publication on DrugGPT along the lines of ChatGPT. This is covered in more detail later in this article**.** In the case of AI-driven drug discovery, GAI may be divided into three classes based on how they are used to generate novel compounds:

• *Distribution-learning* (Brown et al., 2019): The system produces new molecules to fill the same chemical space as the training set. Algorithms for learning distributions are typically judged based on how well they reproduce the characteristics of the training set, with metrics like the Kullback-Leibler (Kullback and Leibler, 1951), (KL) divergence (used, for example, to analyze the distribution of calculated physicochemical features of the molecules) or the Fre’chet ChemNet Distance (Preuer et al., 2018) (FCD), used to quantify structural and functional similarity.
• *Goal-directed generation* (Brown et al., 2019): Molecules are designed in goal-directed generations to optimize some objectives. More specifically, scoring functions refine the generated molecules over time. One method for doing this is reinforcement learning (RL), in which the model is encouraged to pursue strategies with higher likelihoods of success in exchange for a reward. Resemblance to existing active compounds, projected bioactivity, and estimated physicochemical attributes are often used scoring systems (Olivecrona et al., 2017; Blaschke et al., 2020).
• *Conditional generation:* When comparing conditional generation to goal-directed (through a score function) and distribution learning algorithms, molecular generation falls somewhere in the middle. It takes on the challenge of generating new molecules that meet specified criteria by mastering a combined semantic space of attributes and structures found via experimentation. The required qualities may be used as a ‘prompt’ to generate potential molecules. These algorithms enable goal-directed generation without the requirement of scoring function engineering by establishing latent representations covering required properties (such as 3D shape (Skalic et al., 2019a), gene-expression signature (Méndez-Lucio et al., 2020), protein target (Skalic et al., 2019b), and respective molecular structure in an end-to-end manner (for instance, via a conditional RNN (Kotsias et al., 2020). The next section elaborates on various GAI models.

## 3 Generative AI models

**FIGURE 2:**
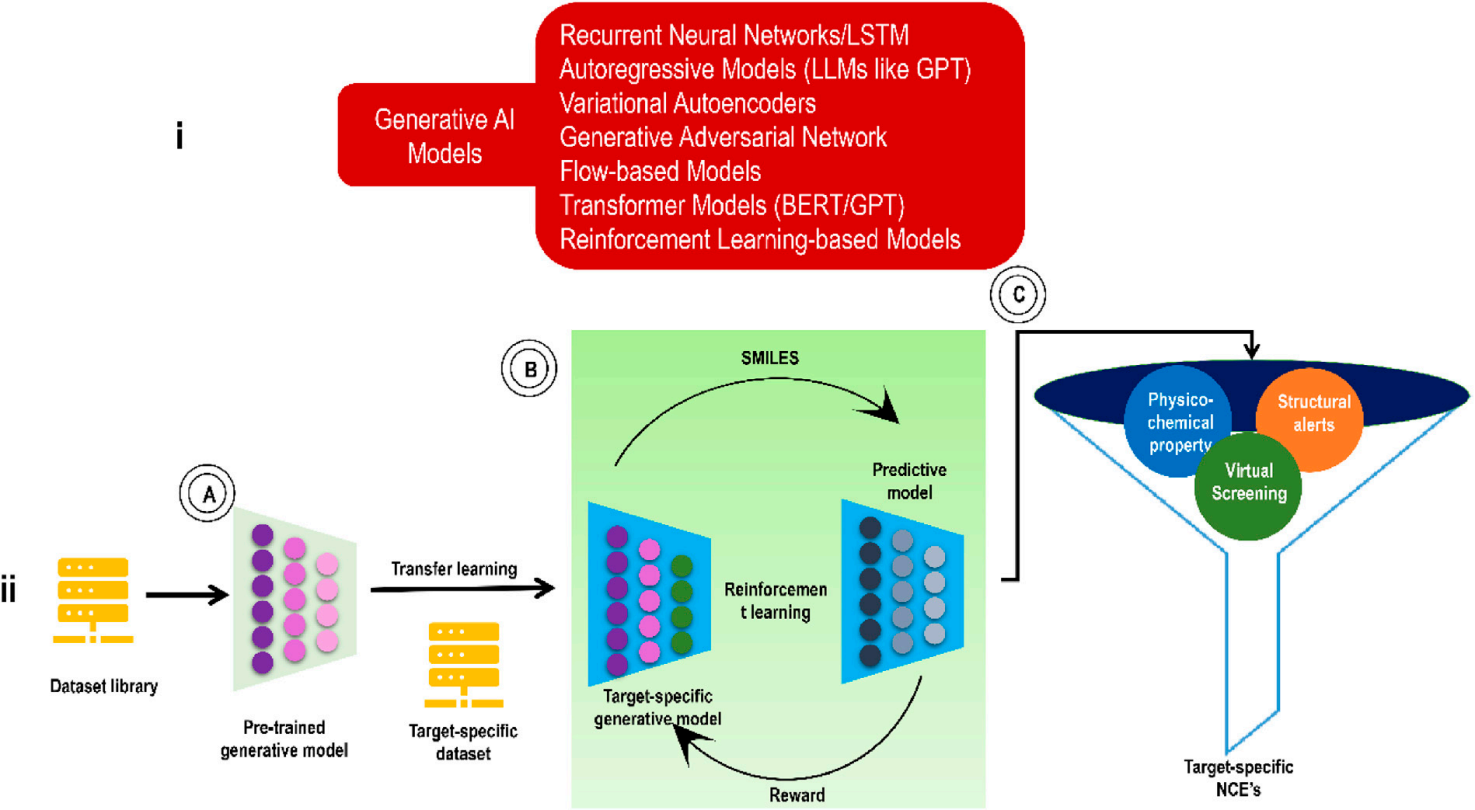
**(i)** Some GAI models; **(ii)** *De novo* molecular design workflow (involving transfer learning to address data scarcity) for generating small molecules against a pre-decided target. **(A)** Pre-trained deep generative model. **(B)** TL to learn the essential characteristics of small compounds concerning the target receptor, and RL approach to enhance the molecular properties in the desired direction. **(C)** Various physicochemical property filters, structural alerts, and virtual screening scores for selecting or ranking model-generated molecules for the next level.

Classifying GAI tools as either non-autoregressive or autoregressive is a common practice. Some examples of non-autoregressive generating models include generative adversarial networks (GANs), reversible flow models, and variational autoencoders (VAEs) (Baillif et al., 2023; Xiao et al., 2023). For example, to construct a graph, non-autoregressive generative models generate both the edge-feature matrix and the node-feature matrix at the same time. On the other hand, iteratively improving an intermediate structure is how autoregressive generative models construct a graph. RNN serves as a prototypical example of the autoregressive generation model. GANs and VAEs are now the most popular GAI models for designing new therapeutic compounds. Transformers and LLMs/Chemical language models (CLMs) are more sophisticated and advanced GAI models. Some of the most fundamental elements of these GAI models include graph neural networks (GNNs), GCN (graph convolutional network)/CNNs (for processing molecular representations in the form of molecular graphs or images), and RNNs for processing molecular representations in the form of sequences like simplified molecular input line entry system (SMILES). In addition, RL is a valuable ML method in the GAI basket. As with the training of robots, driverless automobiles, etc., RL is used to optimize molecular characteristics, such as conditional VAEs and others, to modify the generation of molecules in the desired direction in real-time. In the below subsections, we will see these models individually. Figure 2 shows some GAI models and the general working of a GAI model from the viewpoint of drug discovery via transfer learning.

### 3.1 VAEs

**FIGURE 3:**
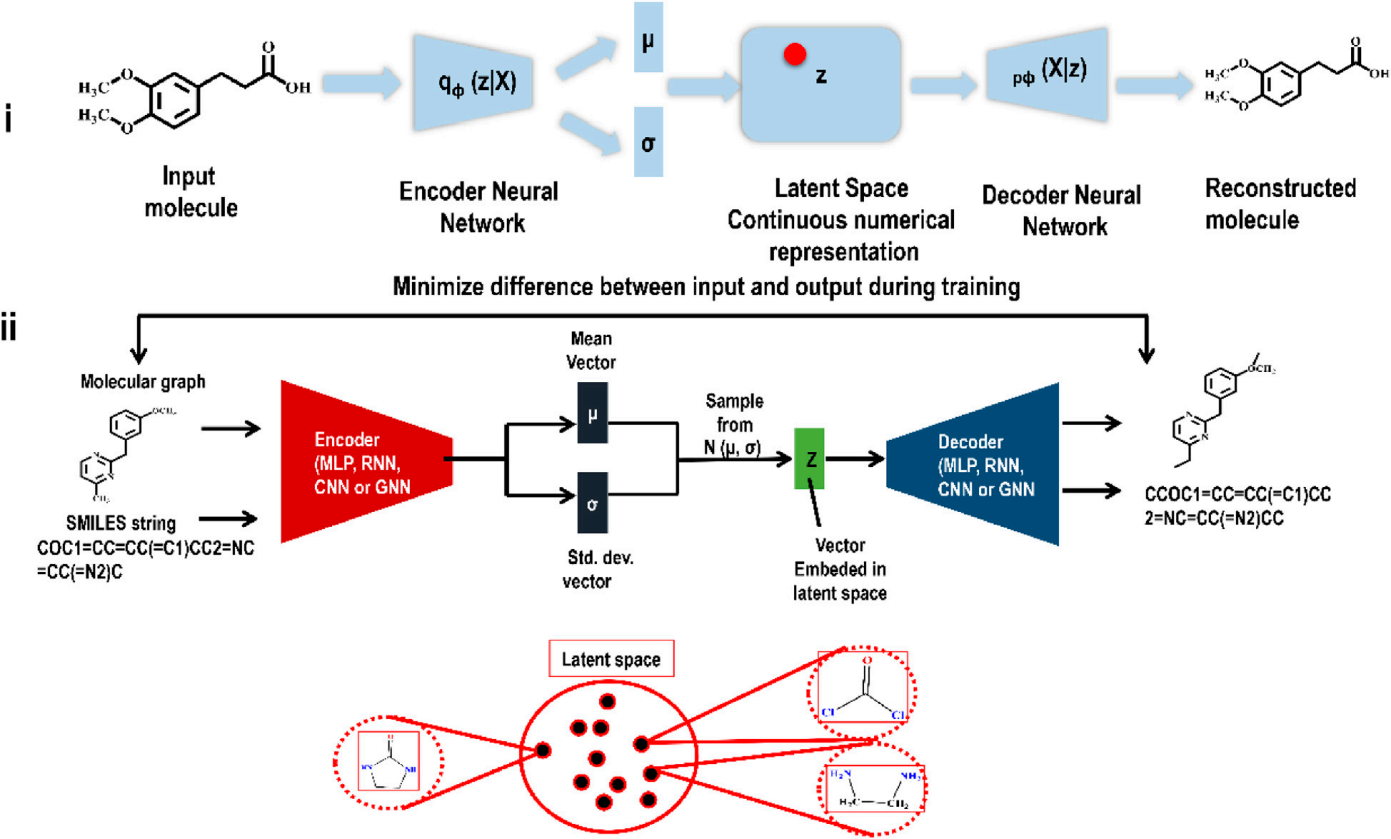
**(i)** Encoding and decoding an input molecule using a VAE. The encoder’s job is to translate a molecule deterministically into a Gaussian distribution. Based on the generated mean (µ) and standard deviation (σ), a novel point is sampled before sending it to the decoder. Finally, the decoder’s job is to generate a novel compound from the sampled point. **(ii)** A detail illustration of a VAE framework indicating how similar molecules lie in latent space.

To build an autoencoder, one must first train a network to map the input into a low-dimensional latent vector through the encoder and then train a second network, the decoder, to map the latent vector back into the input data. By reproducing the input, the autoencoder generates a latent space. However, such basic autoencoders cannot represent molecular structure (in latent space) in a continuous manner. Overfitting and breaks in continuity in the original autoencoders inspired the development of VAEs. Unsupervised data compression using VAEs has become one of the most often used GAI methods due to its success with a wide range of input data types. Many complex data types, including handwritten digits, segmentation, and faces, have been successfully generated using VAEs (Doersch, 2016). VAEs are being used extensively in the search for novel drug-like molecules. In reality, current GAI-based drug development efforts are primarily focused on VAEs. VAEs have two primary components: an encoding network and a decoding network. The encoder network maps the input data to a probability distribution, compressing it into a lower-dimensional representation, the latent space. The input data is reconstructed from a sample taken from the latent space by the decoder network. The VAE seeks to learn optimal parameters to provide the highest likelihood of reproducing the input data. This allows VAEs to learn how to produce realistic samples in the latent space, from which fresh data may be inferred. In the context of drug development, VAEs maximize the similarity between the encoded molecules and a previous distribution in the latent space while minimizing the reconstruction loss during training. VAEs may develop novel molecules with structural and chemical characteristics comparable to the training data by sampling from the learned latent space. Gomez et al.'s research publication on VAE is one of the most often quoted and referred to representative pieces (from the perspective of producing new chemicals) (Gómez-Bombarelli et al., 2018). Several papers followed this seminal work, all of which used VAEs or VAEs after some add-on to generate novel small compounds in the hunt for drug-like molecules. Figure 3 illustrates the functioning of a VAE.

While there are several documented variations of VAE (Blaschke et al., 2018; Harel and Radinsky, 2018; Jørgensen et al., 2018; Kang and Cho, 2018; Sattarov et al., 2019), the essential structure is consistent across all of them. 3D grid-VAE, SMILES-VAE, and Graph-VAE are the three main variants of VAE based on the chemical representations they use. Owing to its simple deployment, SMILES-VAE has gained widespread favour. Additionally, due to their suitability for sequence data structures, stacked GRUs (gated recurrent units) or LSTMs (long short-term memory) are frequently used for both the encoder and decoder in SMILES-VAE. Though effective, the SMILES-VAEs suffer from the same issue that plagues most seq2seq models (namely, the inability to produce 100% verified SMILES strings).

Research groups have shown (Li et al., 2018b; Liu et al., 2018; Samanta et al., 2020; Jin et al., 2023a; Jin et al., 2023b; Jin et al., 2023c), that molecular generators based on molecular graphs can solve the 100% verified SMILES challenge. One such conditional graph generator for multi-objective *de novo* molecule creation was suggested by Li et al. In addition, Jin et al. revealed a string of studies based on Graph-VAE, with the first being a “Junction Tree” VAE. Each node of the junction tree stands in for a component of the original molecule or a single atom. There are two phases involved in making molecules. The first is a scaffolding over chemical components designed as a junction tree. Phase two involves using a graph message-passing network (MPN), a specific kind of GCN, to combine individual chemical building blocks into a whole molecule basis the junction tree (Jin et al., 2023c). Although this approach can keep the molecular representation chemically correct at every stage, the model requires extra work to learn the encoding of the tree structure and decoding the latent variable again to a new tree, making it inefficient for molecular production. Lim et al.'s (Lim et al., 2020), scaffold-based Graph-VAE is another seminal effort in this area; it employs a technique of developing derivative molecules that preserve a specific scaffold as a substructure. The method has the potential to be too particular, rendering it less beneficial when working on a novel, unrelated target protein. Three-dimensional grid-based VAEs, which take cues from 3D image recognition, are the third kind of cutting-edge VAEs (Ji et al., 2012). Inherently, grids make learning from 3D spatial conformations via 3D convolutions easier. This relies heavily on 3D-CNN for both the encoder and decoder. The input data for a 3D grid-VAE model of molecules should likewise be in 3D. However, there is a dearth of readily accessible datasets for 3D Grid-VAE when compared to those utilized for training SMILES-VAE or Graph-VAE. Because bioactive conformations are not always similar to probable least energy conformations, compiling a good 3D molecule database labelled with bioactive data is challenging. Even though some programmes can take 1D or 2D molecular structure data and output it as a random 3D molecular conformation, the actual 3D structure of the molecule, which may be connected to a molecular feature like the IC50 of a particular target, would be lost in the process (Sussman et al., 1998; Wang et al., 2005). These programmes, which are mostly force-field based 3D molecular conformation generators, can only ensure obtaining local potential energy minimum conformations and not the bioactive conformations that have been validated experimentally. In one of the studies, Sunseri et al. (Sunseri and Koes, 2020), designed a helpful toolbox called Libmolgrid, for encoding three-dimensional molecules onto a grid, greatly simplifying the procedure. Libmolgrid employs multidimensional grids to represent atomic coordinates to maintain the spatial connections between the original input’s three dimensions. It also supports contemporary GPU architectures. The atoms are shown as continuous, Gaussian-like densities on a three-dimensional grid with individual channels for every kind of atom. The encoder, made up mostly of 3D convolution networks, takes 3D chemical structures and encodes them into a latent space; the decoder then attempts to decode the latent variable into a new 3D grid display. By expanding the function, which adjusts with the atoms inside the lattice, and connecting the adjusted atoms with suitable geometry, the novel 3D grid may be converted again to a 3D version of a molecule. Although 3Dgrid-VAE shows promise, it has not yet been perhaps used for a genuine drug development effort that includes experimental validation. There have been reports of other AI models for generating molecules in 3D coordinates (Gogineni et al., 2020; Simm et al., 2023).

More and more researchers are paying attention to learning disentangled representations for VAE, where the objective is to have each latent variable in the latent vector capture a different characteristic or aspect of the input (Mita et al., 2023). A molecular feature may be altered independently of other qualities by modifying the latent variables linked with that feature if disentangled VAE is effectively implemented for generating the molecule. In conclusion, there is no ideal framework for generative modelling, but there are good reasons to consider using VAEs. Unlike a standard autoencoder, a VAE’s latent space is continuous and organized, allowing for more precise regulation of produced outputs and more natural interpolation between samples. This is very helpful in fields like drug design, where generating molecules with desired features is essential. In addition, they are more stable and simpler to train than GANs because they have a clearly defined optimization objective and avoid problems like mode collapse (it means the generator will only generate the most probable outputs it has found and will ignore the other modes present in the training data, resulting in a lack of variety in the produced samples.) and training instability. While models like GANs need a large training set to get reasonable results, VAEs may be made to learn in low-data regimes. A successfully produced 3D coordinates may be utilized directly in further molecular computations, including docking, molecular dynamics, and quantum mechanical simulations. We foresee this 3D technology being beneficial for AI-driven drug discovery initiatives in the near future, as evidenced by rapid research and fine-tuning of the models for better results. (Polykovskiy et al., 2018; Kwon et al., 2019; Simonovsky and Komodakis, 2023).

### 3.2 GANs

**FIGURE 4:**
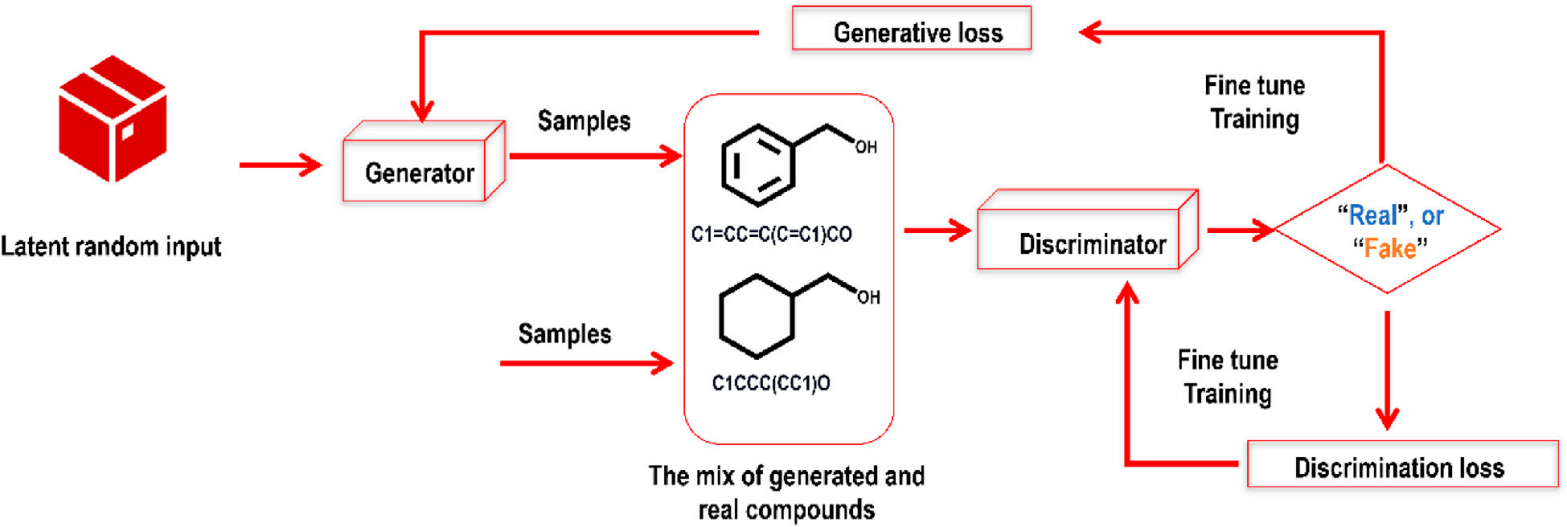
A self-explanatory sample framework of a GAN comprises two modules (the generator and the discriminator) contesting with each other. While training this deep generative model, the generative and discriminative losses are closely monitored.

There has been a surge of new GAI models with the development of GANs (Goodfellow et al., 2020). GANs may be trained to generate synthetic compounds with the required characteristics via an adversarial training procedure. GANs have two parts: a generator and a discriminator. By studying the underlying distribution of actual/real/valid data, GANs may be trained to produce synthetic/fake data such as pictures, movies, and novel molecules. In a drug discovery scenario, expanding the chemical space for prospective drug candidates, GANs may generate novel molecules that are also chemically plausible. The GAN’s generator module takes in noise and outputs a synthetic molecular structure. In contrast, the discriminator module attempts to tell the difference between real and synthetic samples. The generator and the discriminator are trained simultaneously in a competitive fashion, with the generator learning to make more realistic molecules and the discriminator improving its ability to tell them apart. Whereas GANs produce data that is identical to existing data, VAEs generate data that is statistically comparable to existing data. Because of this, they are more suited to activities like designing novel therapeutic compounds with improved characteristics. Figure 4 explains a basic GAN structure.

ORGAN, ORGANIC, and MolGAN (Guimaraes et al., 2017; Sanchez-Lengeling et al., 2017; De Cao and Kipf, 2018), are a few of the GAN approaches that have been documented that use molecular GAN. Both ORGAN and ORGANIC rely heavily on SMILES strings and, therefore, suffer from the same issue of 100% validated SMILES that plagues most seq2seq models. GANs’ training and performance might be erratic and sluggish. Furthermore, training and convergence for GANs are often more challenging. Additionally, for optimal performance, hyperparameters must frequently be fine-tuned. As mentioned earlier, another issue is mode collapse, which is theoretically impossible to prevent (Su, 2018). Regularisation and algorithmic combination may boost their performance. ORGAN, for instance, combines the SeqGAN, as illustrated by Yu et al. (Yu et al., 2016), and the Wasserstein GAN (WGAN) (Weng, 2019), in its design. The team behind ORGAN also tweaked the traditional GAN goal function by including an “objective reinforcement” component in the reward function of generator RNN to encourage the RNN to generate molecules with a specific objective attribute or collection of qualities. Later, building on the foundation laid by ORGAN, the ORGANIC algorithm was suggested to optimize a distribution throughout molecular space in terms of a set of target qualities. Three applications, including organic photovoltaics and melting temperatures, were used to validate the effectiveness of ORGANIC. A graph-based GAN (that uses molecular graphs, unlike ORGAN and ORGANIC) is MolGAN. The computing burden of MolGAN is low, yet it can produce chemically viable structures with 100% accuracy. With the fast acceptance of GANs as one of the most advanced GAI tools for generating novel molecules, their uses in drug discovery are becoming a commonplace. Researchers have used various GAN flavours to aid drug development, and their results have been reported in several distinct studies (Kadurin et al., 2017a; Kadurin et al., 2017b; Putin et al., 2018; Bian et al., 2019; Lin et al., 2020; Bai et al., 2021).

The recent summary of GANs by Tripathi et al. is worth reading and analyzing (Tripathi et al., 2022). In this review, the authors looked at research on drug development that uses several GAN methods to assess molecular *de novo* design. In addition, they explored various GAN frameworks for dimensionality reduction of data during the preclinical phase of drug discovery. They also demonstrated several studies using GAN frameworks in *de novo* peptide and protein synthesis.

### 3.3 Flow-based models

Though they are being used widely, there is no explicit modelling of the true probability density function in either VAE or GAN. To assess the difference between valid (actual) molecules and synthetic molecules, VAE optimizes an implicit lower limit on a probability function, whereas GAN avoids modelling the distribution and instead learns in an adversarial manner. By capitalizing on normalizing flow, deep flow-based models can overcome the intractable problem of explicit density estimation (Rezende and Mohamed, 2023). A normalizing flow is an invertible deterministic transformation between the latent and raw data spaces. For instance, a new technique called MoFlow learns a chain of transformations to map valid molecules to their latent representations and a reverse sequence of transformations to map the latent representations to valid molecules (Zang and Wang, 2006). Shi et al. presented GraphAF to generate graphs using a flow-based autoregressive model (Shi et al., 2021). Using a breadth-first search strategy, they determined the optimal sequence for adding a molecule’s nodes and edges in the training data. The molecular graph generation process was then broken down into parallelizable one-step graph modification procedures. In the generation phase, GraphAF generates a molecule by sampling it repeatedly, a technique that permits the use of chemical domain knowledge in valency testing. Even without chemical knowledge criteria, GraphAF achieved a 68% validity rate on the ZINC data set, which increased to 100% with chemical rules. One significant drawback of flow-based models is the time needed to complete the intricate hyperparameter tuning. Introducing real-value noise into the molecule generation flow may turn the molecular graphs into continuous data, allowing the flow-based models to be used to their full potential. More study is needed in this area to compare these models with prevalent ones.

### 3.4 Diffusion generative models

Diffusion probabilistic models have recently been proven to operate very well across a wide range of generating tasks, and a growing body of research has applied these models directly to the molecule discovery challenge. A series of photos are used to teach a diffusion model. They can learn the statistical connections between pixels in a picture and be used to produce new pictures with comparable characteristics to those in the dataset. One may use these models to get a sequence of interconnected data points. For instance, the Abdul Latif Jameel Clinic for Machine Learning in Health at MIT has developed a novel model called DiffDock that helps hasten drug discovery while decreasing the possibility of side effects. DiffDock employs a diffusion generative model to provide a docking pose space for protein-ligand interactions. More than 100,000 protein-ligand binding postures were used to train this model, all retrieved from the Protein Data Bank-PDB. This technique may uncover possible harmful side effects at an earlier stage in drug development and is more efficient than conventional approaches (as judged by the PDBBind blind docking benchmark, a typical benchmark for measuring the accuracy of molecular docking methods). DiffDock’s potential benefits include lowered drug research costs, faster drug development timelines, and reduced risks to human subjects. DiffDock is still in the developmental phase, but it might significantly alter how drugs are discovered (MITNEW, 2023). Yet another research presents a novel drug development strategy that uses a molecular fragment-based diffusion model. Here, the model may produce compounds with high binding affinity to the target protein and minimal toxicity. The model may produce more varied, valid, and drug-like compounds than other methods. The scientists found the diffusion model based on molecular fragments valuable for finding new drugs (Levy and Rector-Brooks, 2023). There must be more use cases for this strategy in drug development before it can be thoroughly analyzed.

### 3.5 GNNs

Graphs are structured as 2D data representations without any spatial connections between elements. However, it is possible to encode 3D information, such as stereochemistry, into a graph representation. A graph is defined as a tuple G = (V, E) of a set of nodes V and a set of edges E, where each edge e ε E connects pairs of nodes in V. Molecular graphs typically lack directionality, resulting in unordered pairs in E. The concept of the molecular graph representation involves mapping atoms and bonds in a molecule into sets of nodes and edges. One possible approach is to consider the atoms in a molecule as nodes and the bonds as edges. However, alternative mappings are also worth considering. In standard graph representations, nodes are typically depicted as circles or spheres, while edges are represented as lines. In molecular graphs, nodes are commonly represented by letters corresponding to the atom type or by points where the bonds intersect, specifically for carbon atoms. Each node in the graph convolutional layer collects data from its neighbours, representing the regional chemical environment (Bondy and Murty, 1976).

Numerous software packages, for instance can effortlessly visualize the 2D and 3D depictions of graphs, ChemDraw (Reang et al., 2023), Mercury (Macrae et al., 2020), Avogadro (Hanwell et al., 2012), VESTA (Momma and Izumi, 2011), PyMOL (DeLano, 2002), and VMD (Humphrey et al., 1996).

**FIGURE 5:**
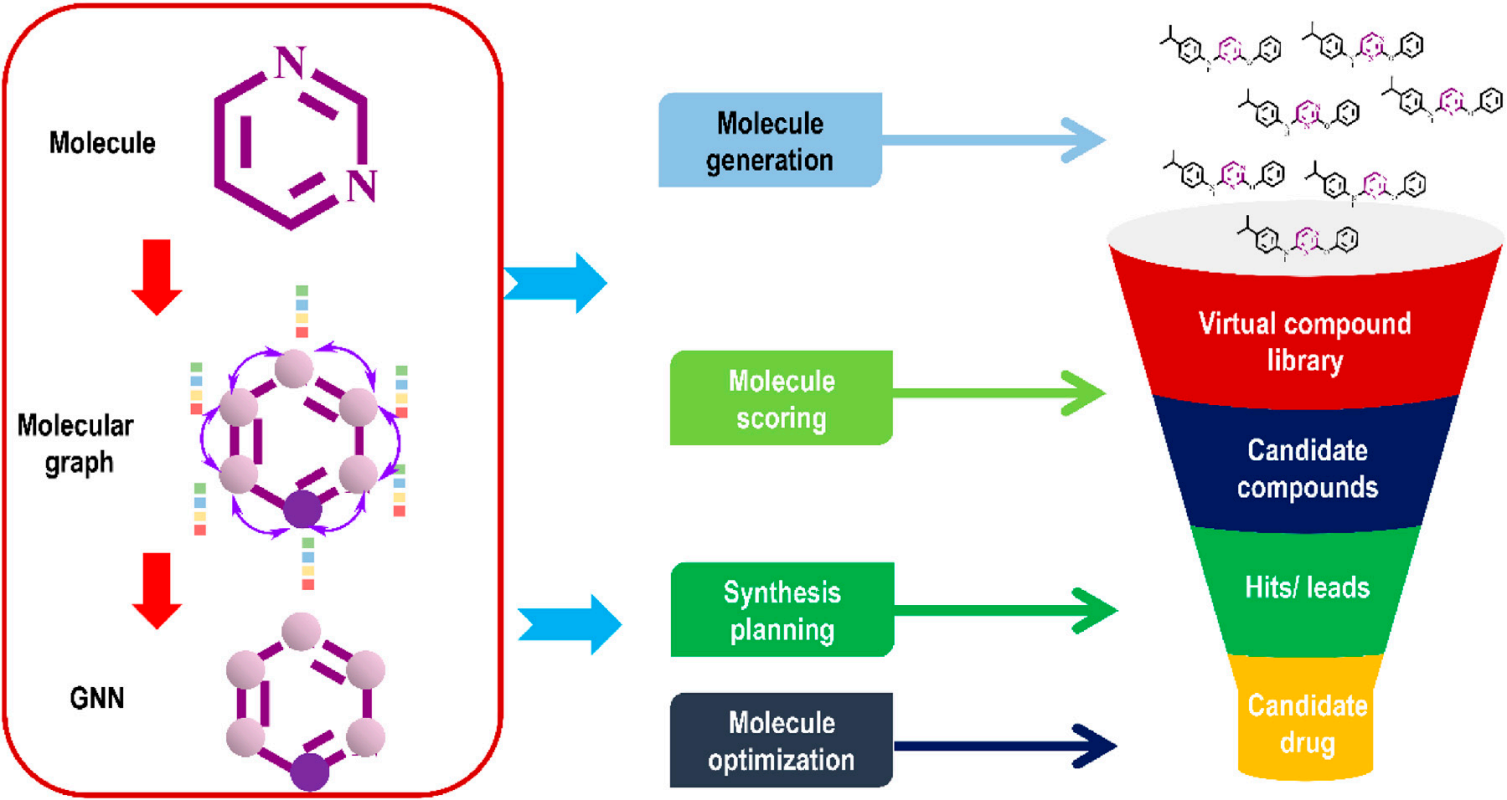
Graph neural networks for automated *de novo* drug design.

Graph-based data, such as molecular graphs, may be directly processed by GNNs. The way these DL models handle molecular inputs makes them advanced neural network. Machines read these molecular representations directly from the molecular graph without requiring extensive modification or engineering by human experts. Among the various varieties of GNNs, the CNN-inspired graph convolution network (GCN) (Wu et al., 2022), and the attention-based graph attention network (GAT) stand out. In a typical molecular graph, atoms serve as nodes and chemical bonds as edges to represent a molecule (Figure 5). To perform tasks such as molecule synthesis, property prediction, and virtual screening, GNNs successfully learn from and generate molecular graphs. To update and disseminate data throughout a graph’s nodes and edges, GNNs use message-passing mechanisms.

Because of their ability to learn association information across heterogeneous and bipartite domains, such as the association between patients and illnesses, GNNs are well suited for generating novel drug-like molecules, predicting their properties, identifying new drug targets, and predicting the interactions between drugs and targets. Studies of GNNs from the perspective of drug discovery and development have been published in recent years by several research groups (Li et al., 2018b; Bradshaw et al., 2019; Lyu et al., 2019; Madhawa et al., 2019).

In their study to predict molecular properties, Jiang et al. showed that GNN was better than other property prediction methods (Jiang et al., 2021). According to recent research, the characteristics of compounds may be reliably predicted using a particular GNN named Chemi-Net (Liu et al., 2019).

While GNNs are among the most cutting-edge approaches to molecular DL setup, they have flaws. For example, GNNs have unique challenges, such as over-smoothing. When too many layers are stacked on top of one another, it becomes impossible to discern between the characteristics of individual nodes in a network, devastatingly affecting model performance. Because of this restriction, modern GNNs typically have no more than four layers. However, as the model becomes more comprehensive, its representational power will grow in tandem. It has been shown that ultradeep neural networks may be helpful in computer vision. Current state-of-the-art CNNs often include over 100 layers (He et al., 2016; Hu et al., 2018). To advance point cloud semantic segmentation, Li et al. (Li et al., 2020a), recently constructed a 56-layer GNN that outperformed prior art by 3.7%. They proved that ultradeep GNN stacking is feasible and showed the benefits of such networks. However, a crucial part of their approach to building such a deep GNN included randomly rearranging the edges of the graph, which does not apply to networks with fixed edges like molecules. As a result, research into techniques for constructing more sophisticated GNNs for molecular learning is also warranted from drug discovery and property prediction.

In addition, numerous types of molecules cannot be adequately described using the graph model. Any structure that includes delocalized bonds, like coordination compounds and molecules with polycentric bonds, ionic bonds, or metal-metal bonds, falls under this category. In the case of molecules with dynamic and ever-changing atomic arrangements in three-dimensional space, the graph representation may lack significance, particularly when bonds between atoms continuously break and form or when the structure undergoes frequent rearrangements. Another challenge in working with graph representations is their lack of compactness. A molecular graph can be represented in various ways, such as an image, a tuple of matrices, lists, or tables. However, these representations are typically more challenging to search through compared to a more concise linear representation, like a string encoding a structure ID. As the size of the graphs increases, they become increasingly burdensome, and their memory usage grows exponentially with the number of nodes (David et al., 2020). The issue does not arise with linear notations, as they utilize the graph framework to generate more condensed and memory-efficient representations for molecules (O Boyle, 2012). Linear notations offer the benefit of being suitable for use as entries in a table and are easily searchable, particularly for identity search rather than substructure search, in cases where a matrix representation is impractical.

Xia et al., in their review, beautifully explained the intricacies of GNNs from the viewpoint of *de novo* drug design (Xia et al., 2019). Another recent study briefly introduced GNNs and their applications in *de novo* drug discovery, including compound scoring, molecule generation and optimisation, and synthesis planning. Readers who need in-depth records may refer to these two review articles (Xiong et al., 2021). For more details on molecular graphs, readers may refer to an exhaustive review article on this subject matter (David et al., 2020).

LLMs have made notable progress in the field of NLP, particularly in terms of their reasoning capabilities. They are primarily intended for the analysis and interpretation of textual data. Nevertheless, there are practical situations in which textual data is linked to intricate graph structures or where graph data is combined with textual information. A thorough investigation by Jin et al. presents a detailed examination of scenarios and techniques pertaining to LLMs on graphs. The study categorizes these scenarios and techniques into three distinct types: pure graphs, text-rich graphs, and text-paired graphs. The paper explores various techniques for utilizing LLMs on graphs, including LLM as Predictor, Encoder, and Aligner. It also provides a comparative analysis of different models, highlighting their respective advantages and disadvantages. The paper also discusses practical applications and includes open-source codes and benchmark datasets (Jin et al., 2023d).

### 3.6 CNNs

**FIGURE 6:**
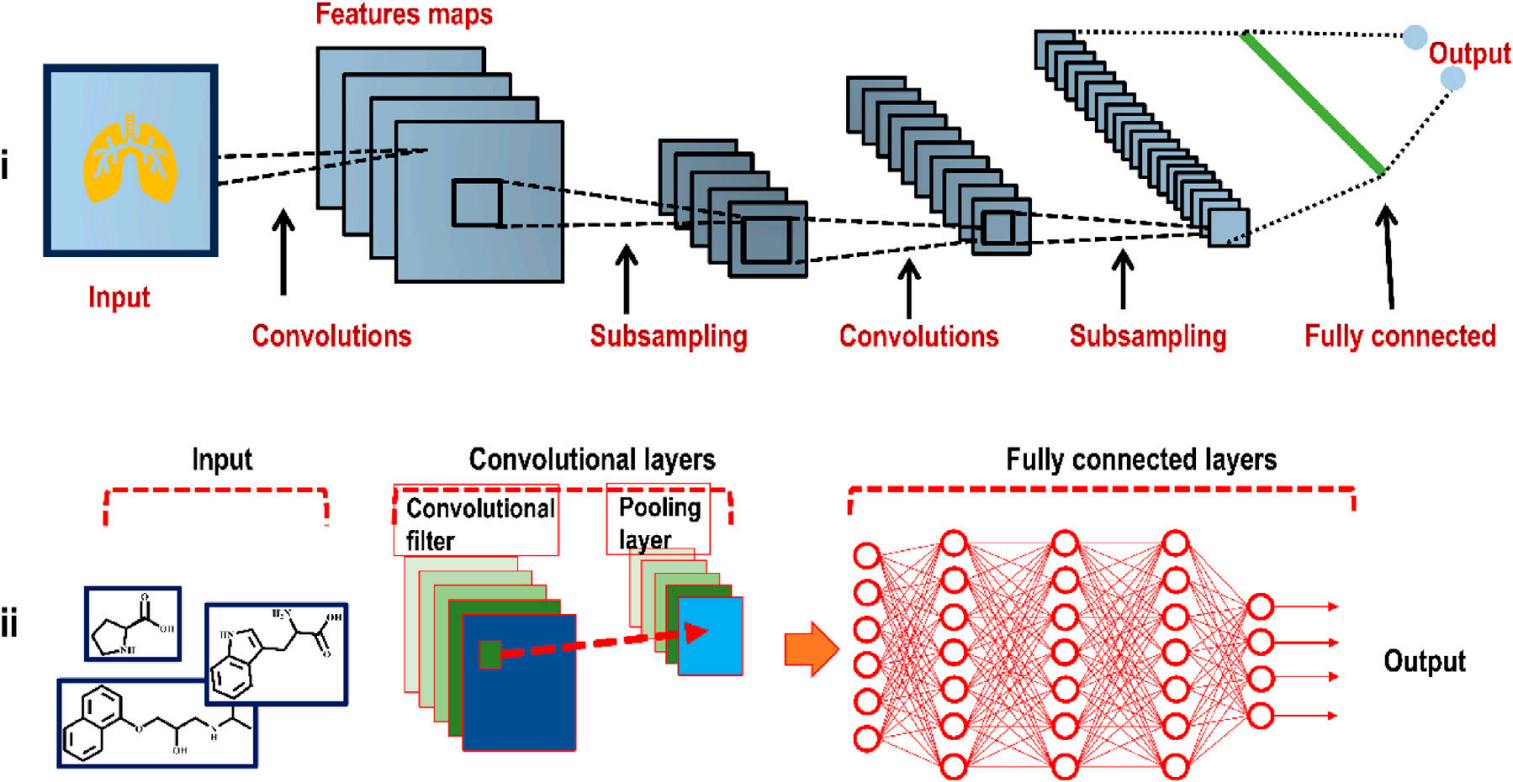
**(i)** Illustration of the basic framework of a plain CNN (deployed to analyze an image, here a heart) consisting of convolutional layers, pooling layers, and a fully connected layer **(ii)** In the case of molecular design, a CNN input includes molecular structures or atom distances from molecular graphs.

CNNs are a sort of artificial network that can automatically extract features from graph or image input by convolutional, pooling, and fully connected layers (LeCun et al., 1998; Rifaioglu et al., 2020; Sun et al., 2020). Running a short window across the input feature vector as a feature detector is a crucial component to the success of CNNs when applied to image processing (LeCun et al., 2015). This method enables a CNN to learn features from the input independently of their location in the feature vector, significantly improving its generalization ability. CNN and 2D molecular structure graphs come together in DeepScaffold (Li et al., 2019) to provide a complete scaffold-based *de novo* drug design solution. A wide variety of scaffold definitions may be used with this approach to build molecules, from cyclic skeletons to Bemis-Murcko scaffolds. One of the benefits of this approach is the ability to generalize the chemical principles for adding bonds and atoms to a given scaffold. By molecular docking of DeepScaffold-generated molecules to their corresponding biological targets, researchers were able to determine whether this method has potential for use in drug development. In another development, DeepGraphMolGen (Townshend et al., 2020) was developed using a graph CNN with RL to generate molecules with desired features. Since 2D graphs are a more natural molecular representation than SMILES strings, they were used in this technique for property prediction and molecular generation. Finally, a novel graph-based approach for drug design from scratch was developed. Figure 6 shows how a CNN works.

The CNN excels with atomistic geometry; hence, it is often used with the voxel (Townshend et al., 2020), (a voxel is a graphic data unit depicting a location in three-dimensional space). There have been several developments involving CNN. For instance, DEEPScreen (Rifaioglu et al., 2020), used a 2D representation of the molecule to predict the drug-target interaction (DTI), while RoseNet (Hassan-Harrirou et al., 2020), AK-score (Kwon et al., 2020), DeepDrug3D (Pu et al., 2019), and DeepPurpose (Huang et al., 2020), each transformed the receptor and ligand into a voxel for the same task. Although DeepConv-DTI (Lee et al., 2019), and transformer-CNN (Karpov et al., 2020), used the CNN framework for sequential input data to develop QSAR models, CNNs are (Karpov et al., 2020), not well suited for sequential expression strategies like SMILES (simplified molecular input line entry system) or protein building blocks.

In multimodal DTI prediction, a discriminative feature depiction of the drug-target pair plays a key role. Dehghan et al. proposed a novel multimodal method called TripletMultiDTI that leverages triplet loss and task prediction loss to accomplish this goal. This method provides a new framework that fuses the multimodal knowledge to predict interaction affinity labels. TripletMultiDTI also offers a novel loss function based on the triplet loss to learn more discriminative depiction. Using this model, researchers improved prediction results, as evident from the proposed approach’s evaluation on three putative datasets (Dehghan et al., 2023).

Palhamkhani et al. introduced DeepCompoundNet, a sophisticated model that employs DL to combine protein characteristics, drug properties, and various interaction data to predict chemical-protein interactions. This novel model surpassed the most advanced techniques in predicting compound-protein interactions, as evidenced by performance evaluations. The results of this study emphasize the synergistic relationship between various interaction data, which goes beyond the similarities in amino acid sequence and chemical structure. DeepCompoundNet achieved superior performance in predicting interactions between proteins and compounds that were not detected in the training samples (Palhamkhani et al., 2023).

### 3.7 RNNs

The RNN is the most basic model for molecular GAI models. Perhaps the first team to use RNNs to generate compounds was Bjerrum et al. [ (Bjerrum and Threlfall, 2017). The concept came straight from the AI’s NLP subfield (Chowdhary and Chowdhary, 2020). The process of generating new molecules is recast as the creation of new, unique sequences of characters. These similar sequence models are sometimes called “seq2seq” (Sriram et al., 2017), because both the input and the output are sequences. Sequences from high-dimensional molecules are first reduced to a single dimension in the seq2seq procedure. Many programmes can do this. Owing to its long usage history and human legibility (Tang et al., 2020), the simplified molecular-input line-entry system (SMILES) is among the most used sequence string pattern for describing chemical structures. By modelling the molecule generation process as a series of phases (like phrase construction in a language translation job) and sampling from the network at each stage, it is possible to generate molecules that are likely genuine and chemically equivalent or improved versions of the training compounds (Olurotimi, 1994). An RNN may acquire the common associations between chemical building blocks after training on a known-molecule database. After training, the network can predict the degree of similarity between two atoms or functional groups. These probabilities will shift depending on whatever portion of the molecule the network has seen before. Attaching an extra model (like RL) as a property optimizer also yields unique compounds. Each input value impacts the subsequent output value when sequential data are fed into the RNN step by step.

**FIGURE 7:**
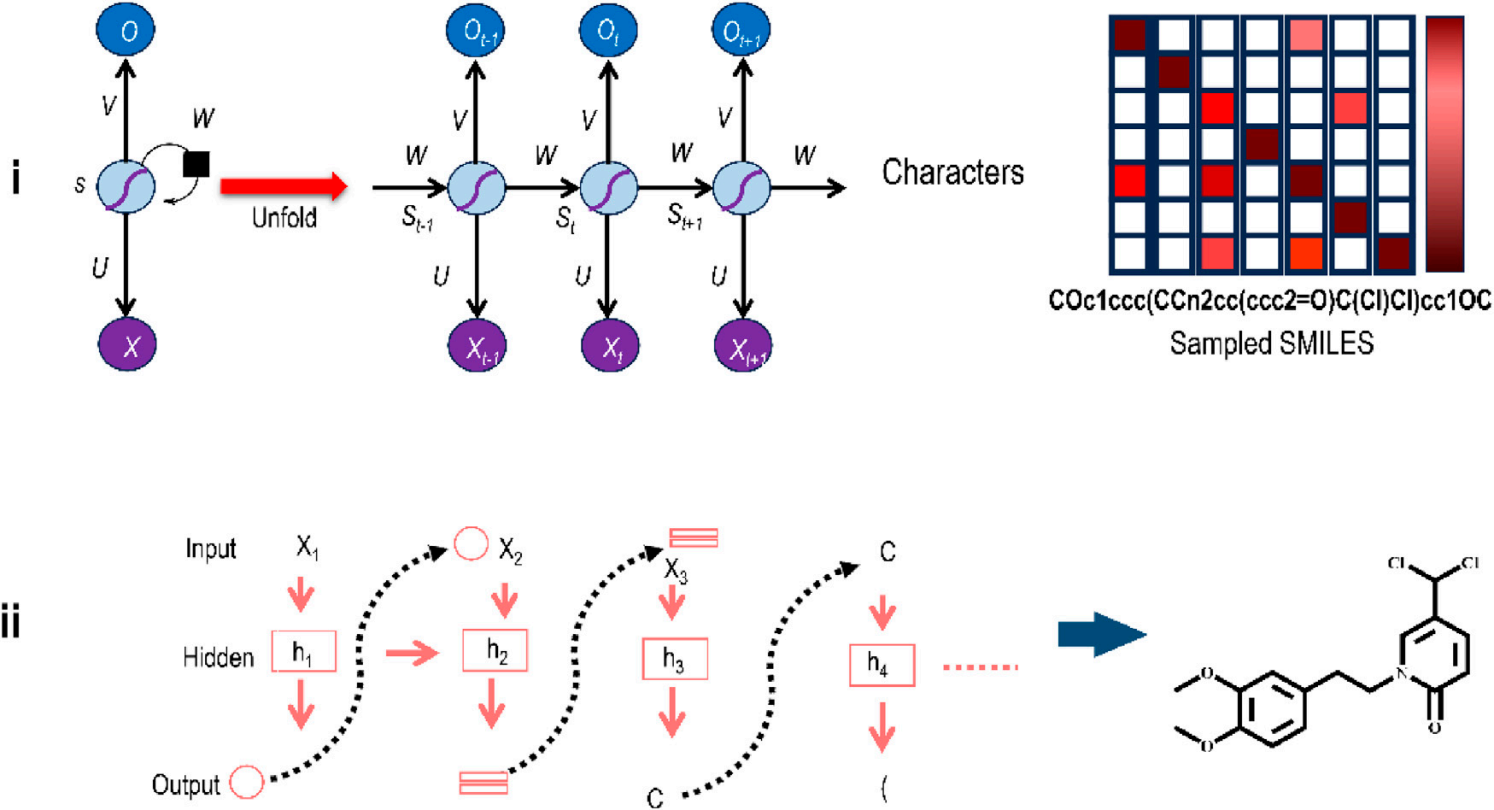
**(i)** The elementary framework of an RNN showing input unit (x), hidden unit (s), and output unit (o). Different weight matrixes are U (for the shift from x to s), V (for the shift from s to s), and W (for the shift from s to o). **(ii)** Structure generation from RNNs as part of DL-driven *de novo* molecular design in a sequential manner.

Figure 7i illustrates an elementary RNN framework. The left side of this illustration indicates a folded RNN with a self-loop, which means the hidden layer s is used to modify itself depending on the input x. The right side of the illustration shows an unfolded RNN as a sequential framework to show its working. Sequential data x1, x2, . . , xt are fed to the RNN as input values, where at every step t, xt is a d-dimensional feature vector. For instance, if the model gets a group of words as input, then every word wi is shown as a vector xi. At every time-step t, the result from the preceding step, st-1, with the subsequent word vector in the document, xt, are leveraged to modify the hidden state st as st = f(Wst-1+Uxt) where f is the nonlinear activation function and U and W are the weights of inputs xt, and st-1 respectively. st, the hidden state, is the feature depiction of the sequence up to time t for the sequential input data. The opening states s0 are typically started as all 0. Thus, one can use it to accomplish various assignments like sentence building, document cataloguing, etc.

A similar idea may be used for generating molecules, as shown in Figure 7ii. The upper part of this Figure shows how an RNN applies logic to handle and process the information to generate the structure (right bottom). The *y*-axis displays all potential tokens that may be picked at each step; the color reflects the conditional probability for the character to be chosen at the current stage considering the previously picked characters, and the *x*-axis displays the character that was sampled in this case. The actual working of an RNN in structure-generation mode is shown in the bottom left part of the Figure. At every stage, a character is sampled based on the conditional probability distribution derived from the RNN, and the newly generated character will be utilized as the input for the next character to be generated.

Nonetheless, the fundamental RNN has a not-so-complex structure and has performance restrictions for uses in diverse contexts. The vanishing gradient problem is the most pressing issue. As the size of the input series increases, the influence of things distant from the presently inputted item decreases exponentially, resulting in low performance for lengthy data like proteins and other big molecules (Hochreiter, 1998). Moreover, training time is proportional to sequence length since the same operation is performed many times. Even in cases where the elements in sequential data have intricate associations, the features of these elements are not effectively learned. To address this vanishing gradient problem (which prevents the weights from being updated), the theory of long short-term memory (LSTM) was developed years ago and recently adopted extensively (Yu et al., 2019). In contrast to standard RNNs, the LSTM still performs well on longer data sequences. Several LSTM variants have been developed since its inception, and lately, gated recurrent units (GRU) with a simplified internal framework have also seen widespread use (Cho et al., 2014a; Mouchlis et al., 2021). Feeding a target protein sequence may be utilized for *de novo* drug discovery by arbitrarily generating small molecules (Jastrzębski et al., 2016). The LSTM and GRU outperformed the conventional RNN and are commonly used in drug discovery; however, there is still scope for newer methods to address the vanishing problem more effectively, especially for very long sequence data (Guimaraes et al., 2017; Popova et al., 2018; Martinelli, 2022). Validity, novelty, and variety improved when RL was coupled with a stacked RNN (Olivecrona et al., 2017; Gupta et al., 2018; Segler et al., 2018; van Deursen et al., 2020).

### 3.8 Reinforcement learning

**FIGURE 8:**
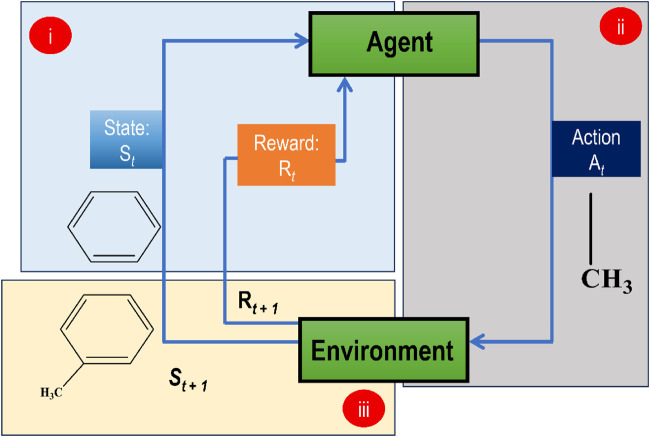
Demonstration of a basic RL model for *de novo* molecular design **(i)** A benzene ring at state St at iteration t, and an initial reward Rt at iteration t, **(ii)** the agent picks an action At which attaches a methyl group to benzene **(iii)** The environment takes this knowledge for generating a subsequent response that is St+1 state and a new reward Rt+1. This sequence of events goes on until the episode ends.

In RL, an agent learns to make successive choices in an environment to maximize a cumulative reward signal. In drug development, RL may optimize molecular characteristics in real-time throughout the design process if coupled with a GAI model. A drug discovery RL agent interacts with a representation of the chemical space, and at each step, it chooses an action (such as changing a molecule structure) that best fits its current situation. The generated molecule’s projected activity, binding affinity, and other attributes provide a reward signal to the agent. In this way, an RL algorithm, coupled with a GAI model, may generate compounds with desired qualities or optimize particular molecular attributes by iteratively exploring and utilizing the chemical space. Figure 8 indicates how an RL approach is used for generating optimized molecules.

Optimizing an agent’s ability to exert influence over its environment is made possible by RL theory, which offers a normative explanation based on psychological and neuroscientific views of animal behaviour (Mnih et al., 2015). The RL algorithm ensures that the GAI model is always optimized by constantly interacting with the environment and adopting actions to maximize predicted cumulative rewards. Unlike supervised and unsupervised ML, in which performance is limited by the availability of human previous knowledge and training data, RL is unconstrained by these factors and may provide superior results. The method may be used for various settings and issues (Chen, 2016; Li and Du, 2018; Zhu et al., 2018; Holcomb et al., 2019; Vázquez-Canteli and Nagy, 2019). AlphaGo is the most well-known application of RL algorithm ever since it beat human Go champions. REINVENT, developed by AstraZeneca researchers, is perhaps the first study linked to an RL-based model (Olivecrona et al., 2017), for applications in drug development. Drugs against dopamine D2 receptor, and analogues of the COX2 inhibitor, celecoxib, were generated, and their activities were predicted using this REINVENT. In developing REINVENT, a basic generative model based on RNNs was trained first; then, it used the RNN that had already been trained as a model for the agent network (Pal et al., 2018).

ReLeaSE (Popova et al., 2018), is another RL-based RNN model that uses a Markov decision process (MDP) to describe the generation of SMILES strings. It has fundamental RL components. In contrast to REINVENT, ReLeaSE employs a fully connected network prediction model as a reward function, which also requires pre-training. Both generative and predictive networks are crucial to ReLeaSE’s success. The authors behind ReLeaSE showed that ReLeaSE may be used to produce a library of JAK2 inhibitors. It may be challenging for the RNN to generate bioactive SMILES if the predictor is not trained appropriately. Motivated by the findings presented by Mnih et al., in 2015 (Mnih et al., 2015), Zhou et al. (Zhou et al., 2019), developed a generative model called Molecule Deep Q-Networks (MolDQN) by combining RL with chemical rules to circumvent issues with SMILES-based RL models. MolDQN guarantees complete chemical validity by specifying alterations to molecules in terms of adding or removing specific atoms or chemical bonds. Because MolDQN does not need specific training data, it is immune to biases introduced by the dataset on which it is used. The MolDQN model differs from the seq2seq models in that it describes the alteration of a compound as a molecular Markov decision process (MDP) (Van Otterlo and Wiering, 2012), and employs the deep Q-Networks (DQN) (Hester et al., 2017), to answer this MDP with the essential attributes as rewards. However, MolDQN’s performance while generating molecules is subpar. The stated modification only contains three distinct kinds (atom addition, bond removal, and bond addition); hence, it takes at least six steps for MolDQN to build a benzene molecule, for example,. All generated compounds have chemical significance; however, they may be too challenging to synthesize or have unfavourable drug-like qualities.

Covalent inhibitors to take on the SARS-CoV-2 major protease (a significant therapeutic target for COVID-19) were developed by Tang et al., in 2020 using an advanced deep Q-learning network with fragment-based drug design (ADQN-FBDD) (Tang et al., 2022). ADQN-FBDD is more effective at building molecules than MolDQN since it uses fragments rather than individual atoms. The DQN agent, guided by the reward function, picks out rewarding pieces to affix to the right spots in the current state (the intermediate structure). ADQN-FBDD generates more drug-like compounds because it considers chemical reaction laws and the three-dimensional shape of the binding site. As a result, ADQN-FBDD can effectively probe the chemical space around the given target.

The capacity of RL-based models to perform distributed computations is their greatest strength since this drastically shortens training times in many scenarios. When several computers are deployed, even the vast chemical space may be investigated efficiently (Horgan et al., 2018). However, the quality of the representation of the training data might significantly impact the performance of the models mentioned above. In contrast to efficient representations, which capture the most relevant information, ineffective representations lose key aspects while training AI models. Exciting new AI algorithms are being studied to fill this gap. For instance, researchers merged RL with variational inference in one of the studies to get better results (Fellows et al., 2019).

### 3.9 Transformer

Relevance and meaning may be taught using a specific type of GAI called a transformer by tracing the links between sequential data, like the words in this statement. Transformer models apply to any input data as long as they use sequential text, picture, or video data. A transformer is used, for instance, whenever a person searches for something on search engine like Google, Bing, etc. GPT-4, BERT (Bidirectional Encoder Representations from Transformers), RoBERTa, XLNet, and Megatron-Turing NLG are all examples of transformers. To find how seemingly unrelated data points in a sequence impact and rely on one another, transformer models are used by leveraging a growing body of mathematical techniques collectively referred to as attention or self-attention. Transformers, first detailed in a Google study published in 2017 (Vaswani et al., 2017), are cutting-edge and highly effective GAI models. Nowadays, text and voice are being translated in real time by transformers, allowing a wider range of people, including those with hearing impairments, to participate in meetings and classes. In the medical field, transformers are being tried to assist scientists in deciphering gene and protein chains to expedite drug development. Technically, transformer models are huge encoding/decoding building components like most neural networks. The special power of transformers comes from the careful arrangement of their building components. Transformers use positional encoders to assign tags to data packets entering and leaving the network. The attention units then use these tags to create an algebraic map of the relationships between the various components. In multi-headed attention, attention inquiries are often conducted in parallel by computing a matrix of equations. Computers can now recognize patterns just as humans can with the help of these programmes (Monteiro et al., 2022).

The most common forms of DL only 5 years ago—CNNs and RNNs Networks—are being supplanted by Transformers. Seventy percent of the AI studies submitted to arXiv in the last 2 years make reference to transformers. This contradicts the findings of an IEEE research from 2017 that found RNNs and CNNs to be the most widely used models for pattern recognition. Before transformers were designed, users had to train neural networks to create large, labelled datasets, which was time-consuming and costly. Transformers eliminate this necessity by discovering mathematical patterns between portions of the billions of pictures and petabytes of text material stored in the internet and business databases. In addition, transformer mathematics is well-suited for parallel processing; therefore, these models are quick to execute. Popular performance leaderboards like SuperGLUE, a benchmark created in 2019 for language-processing systems, are now dominated by Transformers (Dosovitskiy et al., 2020).

A year later, another Google team tried processing text sequences both forward and backward with a transformer. This enhanced the model’s capacity to comprehend the meaning of a phrase by capturing more links between words. Their BERT model broke 11 records and was included in the Google search algorithm. Text is one of the most typical data kinds that businesses have; therefore, within weeks, researchers all across the globe were modifying BERT for use cases across various languages and sectors. BERT is especially adept at comprehending the context of words in a phrase since it was trained on a large text sample. A few activities that BERT is helpful for are sentiment analysis, named entity identification, and question-answering. Most information in the pharmaceutical industry is based on the structures of small molecules and comparatively larger molecules (receptors, amino acids, antibodies, etc.). The representation of these structures may take the form of strings or other sequences. For instance, DeepMind (an Alphabet-owned company) used a transformer known as AlphaFold2 to increase our knowledge of proteins. It processed amino acid sequences like text strings to establish a new watermark for characterizing how proteins fold. In another development, AstraZeneca and NVIDIA created MegaMolBART, a transformer specifically designed for drug discovery. It is a scaled-up version of the MolBART transformer developed by the pharmaceutical industry and trained on a sizable, unlabeled library of chemical compounds using the NVIDIA Megatron framework for creating large-scale transformer models. The academic health centre at the University of Florida also worked with NVIDIA researchers to develop GatorTron. This transformer model seeks to speed medical research by gleaning insights from vast clinical data (Devlin et al., 2018).

For generating drug-like compounds, Bagal et al. trained a transformer-decoder on the next token prediction task with masked self-attention, inspired by GPT models’ success in producing meaningful text. In terms of producing valid, unique, and innovative compounds, their model, dubbed MolGPT, performed on par with other recently suggested current ML frameworks for molecular generation. They also showed that the model may be taught conditionally to influence specific characteristics of the synthetic compounds. MolGPT may be utilized to build molecules with the desired scaffolds and property values by conditioning the molecule generation on scaffold SMILES strings with required scaffold chemical attributes (Bagal et al., 2021).

A recent finding emphasizes the growing interest in exploring bioactive molecules in cancer cell lines. The study proposes a novel DL-based approach called DeepTraSynergy for predicting drug combination synergy, recognizing the enhanced efficacy of multidisciplinary drugs in cancer treatment. The approach utilizes transformers to learn feature representations of drugs and incorporates multimodal input, including protein–protein interaction, drug–target interaction, and cell–target interaction. DeepTraSynergy employs a multitask strategy, predicting three outputs: toxic effect, drug–receptor interaction, and drug combination synergy, with synergy being the primary task. Three loss functions are defined:, toxic loss, synergy loss, and drug–protein interaction loss. DeepTraSynergy surpasses conventional and advanced models in predicting synergistic drug combinations on DrugCombDB and Oncology-Screen datasets, achieving accuracy values of 0.7715 and 0.8052, respectively. The evaluation of each component of DeepTraSynergy demonstrates its effectiveness, particularly highlighting the significance of incorporating protein–protein interaction networks in improving the prediction of synergistic drug combinations (Rafiei et al., 2023).

In conclusion, the attention technique analyses historical data concurrently, enabling the application of correlation with distant tokens without reduction, in contrast to the RNN, where only one hidden state is available. Furthermore, BERT have been used in drug discovery endeavors and have greatly improved natural language presentation by employing DL (Devlin et al., 2018). For DTI applications, the transformer model may be easily included in the prevalent RNN-based QSAR modelling. Pharmacological action predictions were made by Karpov et al. (Li et al., 2019) using a model that applied CNN to a transformer and used as input SMILES strings. For instance, by combining the protein sequence with CNN and the chemical structure with BERT, researchers proposed a molecular transformer DTI (MT-DTI) technique to estimate ligand-receptor binding affinity (Shin et al., 2023). In a similar vein to MT-DTI, but using BERT for protein and molecular structures, another group of researchers proposed a GCN-based method (Lennox et al., 2021). The goal of the transformer (in the context of chemical compounds) is to learn the connections between atoms, just as LLMs may learn the links between words in a sentence.

### 3.10 LLMs and CLMs

As was previously noted, the groundbreaking product ChatGPT is making inroads, thanks to LLMs. Such LLMs are often constructed with the help of transformers. The latest version of ChatGPT has significantly increased interest in LLMs. It should be noted, however, that LLMs had already made substantial contributions in fields like voice recognition, machine translation, and part-of-speech tagging long before this. Hundreds of millions of users utilize ChatGPT, and there are other systems like Google’s Bard. Such systems have a straightforward method of operation. The user inputs a query, and the AI system responds with a string of words (technically tokens) in an autoregressive fashion, with each word feeding into the prompt for the next word’s generation.

**FIGURE 9:**
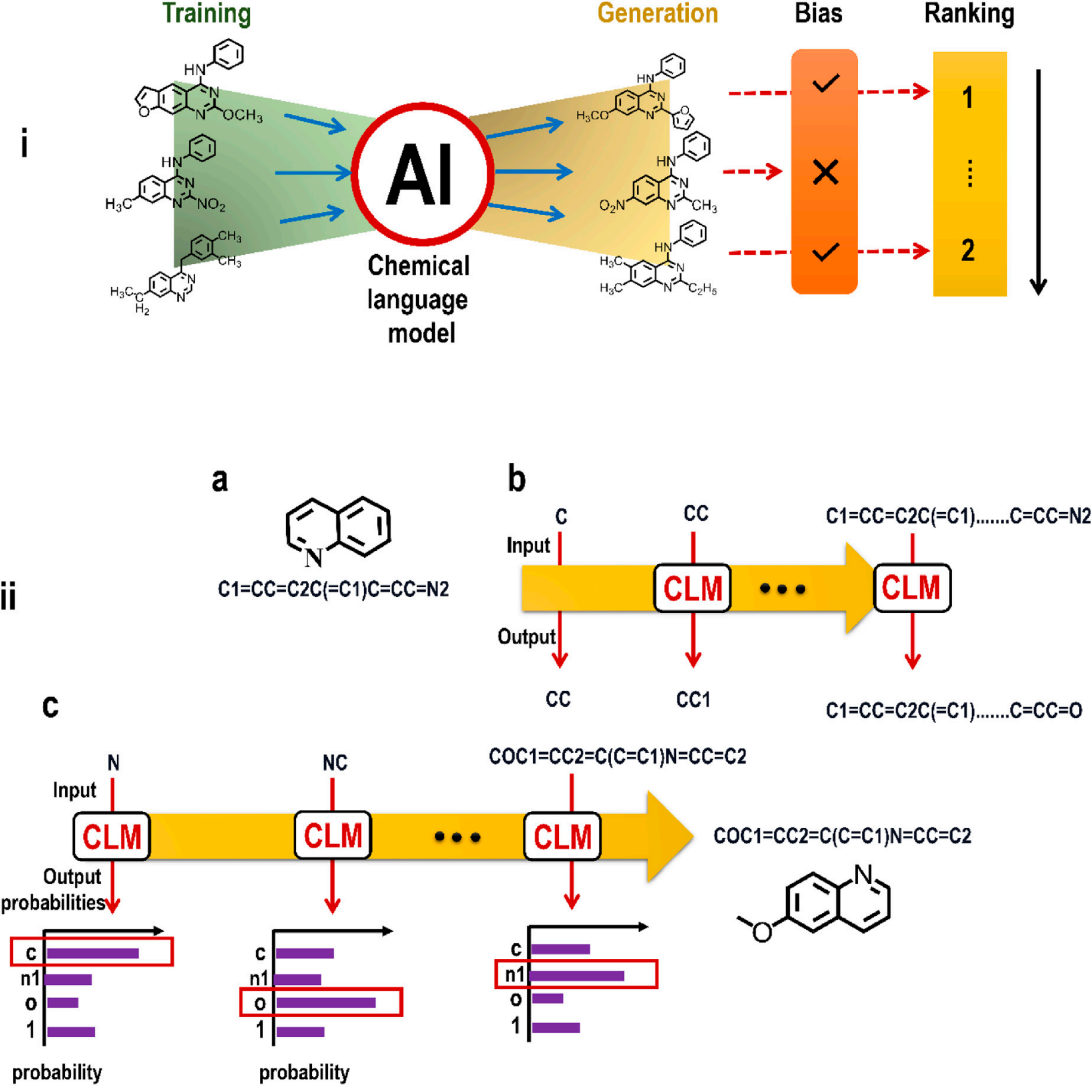
**(i)** An illustration showing the working of CLM on the basic framework of LLM; **(ii)** Principles of chemical language models (CLMs). **(a)** Example of a molecular structure (Kekulé structure) and a corresponding SMILES string. **(b)** CLMs are trained to iteratively predict the next SMILES character based on the preceding string characters. **(c)** Multinomial sampling can be used to generate new SMILES strings from trained CLMs, where SMILES characters are sampled with a weighted random sampling of probability distributions learned by the CLM.

Specialized transformers with hundreds of billions or, more recently, trillions of computable parameters exist behind the hood of an LLM. This model is then trained on enormous datasets taken from digitally accessible human-generated text such as that found on the internet. The cost of training such a huge model is high because it requires the employment of thousands of specialized pieces of computing hardware called graphics processing units (GPUs) to do the necessary trillions of mathematical calculations. The next-generation LLM GPT4 is more capacious and proficient in text and picture processing than its predecessors. It is probably not an exaggeration to state that LLMs like ChatGPT have effectively split times into two parts: before and after ChatGPT. Such models will undoubtedly inspire and inform future upgrades and advancements in GAI-driven drug development. The idea of analyzing sequence data and then producing new sequences (based on the grammar of these data) has been met with tremendous success, and it is currently being applied to chemical structures. In this sense, molecules may be seen as the building blocks of a chemical language (Figure 9) (Bralley, 1996).

There is a prescribed grammar for chemically valid compounds, just as in human language, in which discrete components (atoms, like words) may link (form bonds) only in specific ways. Different high-level features (such as physicochemical, biological, and toxicological) arise from the presence and arrangement of molecules’ constituent parts, giving rise to the concept of “semantic properties. *De novo* drug design (Elton et al., 2019), tackles the challenging topic of how to construct compounds afresh that are chemically valid (that is, syntax) and contain desirable pharmacokinetic and pharmacodynamic qualities (that is, semantics), making knowledge of the chemical language essential. The chemical cosmos that *de novo* designers must work with is enormous, with as many as 10^60^ tiny molecular entities to consider (Bohacek et al., 1996). This number is so high that it would be impractical to attempt a comprehensive enumeration. In particular, GAI has benefited *de novo* drug design domain from the current AI revival (in the form of DL) (LeCun et al., 2015)**.** Chemical language models (CLMs) (Yuan et al., 2017; Segler et al., 2018), have been at the forefront of AI-driven *de novo* design. Since CLMs may theoretically synthesize many molecules in a single shot without expert-engineered rules, they show significant potential for navigating chemical space and exploring sparsely occupied places (Skinnider et al., 2021; Flam-Shepherd et al., 2022). CLMs use algorithms already established for NLP to understand chemical language. Simplified Molecular Input Line Entry Systems (SMILES (Weininger, 1988)), strings are an example of a string notation that enables this. Bioactive substances generated by CLMs and verified in investigations (Yuan et al., 2017; Merk et al., 2018b; Moret et al., 2021), show that this class of GAI may explore uncharted biochemical areas. DL algorithms have spurred a revival (Wiswesser, 1985; Öztürk et al., 2020), in using sequential molecular representations, initially developed for storing large databases and identifying molecules in the sequence processing field. The most popular molecular string representations for *de novo* design are:

• *Simplified Molecular Input Line Entry Systems (SMILES)* (Weininger, 1988)**:** SMILES strings are created by transforming H-depleted molecular graphs into a series where respective atomic symbols signify the atoms, symbols represent the bonds and branching, and numbers represent the opening and closing of rings. SMILES are not unambiguous since they may be found by travelling through the molecular graph in any direction and starting from any non-H atom. Canonicalization procedures are required to produce a univocal SMILES string (Weininger et al., 1989; O Boyle, 2012). The use of numerous SMILES to represent the same molecule (Bjerrum, 2017; Arús-Pous et al., 2019), for artificially growing the number of samples (in case of inadequate data) to train CLM, commonly known as data augmentation, has been proven to have positive effects in several investigations. Data augmentation is a method for making a dataset seem larger than it is.
• *DeepSMILES*: To fix erroneous syntax caused by imbalanced parentheses and ring closure pairs, DeepSMILES (O Boyle and Dalke, 2018), was suggested to enhance SMILES. Applications of DeepSMILES for predicting drug-target binding affinity (Öztürk et al., 2019), have been made, although the complexity of its syntax makes it less amenable to molecule creation than SMILES strings (Arús-Pous et al., 2019).
• *Self-referencing Embedded strings (SELFIES)* (Krenn et al., 2020): SELFIES are constructed from semantically limited graphs, allowing each symbol to be utilized to generate a different graph from the series. Every SELFIES string, unlike SMILES, is equivalent to a legitimate chemical graph. Though most SELFIES strings are valid, in certain circumstances, the validity is assured by *post hoc* string shortening (Gao et al., 2022).

Every representation may be seen as a separate chemical language with its own grammatical conventions that must be followed to produce chemically sound molecular entities. It has been noted that SELFIES can avoid the requirement to learn chemical grammar (since these strings always match genuine molecules) (Krenn et al., 2022). According to recent research, knowing the syntax of SMILES strings makes identifying and maintaining *de novo* designs that fit the target chemical space easier than SELFIES (Skinnider et al., 2021). This aligns with research in the NLP field (Russin et al., 2019), which emphasizes the advantages of syntax learning to provide improved semantical features. Overall, it seems that the improved performance of SMILES, SELFIES, or DeepSMILES is application-dependent, with generally modest differences (Chithrananda et al., 2020; Skinnider et al., 2021). InChI representations (explaining chemical compounds through layers of information separated by "/") were also employed with CLMs; however, their performance was very poor compared to SMILES. Compared to the complexity of structures like graphs, the simplicity of text generation makes molecular strings an appropriate representation for molecule generation. However, linear notations have other limitations, such as that atoms close together in the molecular graph may be far apart in the equivalent string (because of rings and branches). This may be the case since CLMs have been demonstrated to benefit from both bidirectional learning procedures (Flam-Shepherd et al., 2022) and the incorporation of language information (Kusner et al., 2017; Grisoni et al., 2020). Many flavours of GAI have been used for chemical language modelling (Liu et al., 2020; Öztürk et al., 2020).

Similarly, RNNs with memory cells (Hochreiter and Schmidhuber, 1997; Cho et al., 2014b), have found widespread usage (Brown et al., 2019; Polykovskiy et al., 2020; Flam-Shepherd et al., 2022), in drug discovery research. RNNs are often taught to create a single character at a time, with each character being informed by the characters that came before it in the molecular string. This allows them to be used to generate new molecular strings (Suresh et al., 2022). Other popular CLM architectures, as seen earlier in this review, are a) VAE, constituted by an encoder that converts molecular strings to latent vectors and a decoder that converts latent vectors back to molecular strings, and b) GANs, constituted by a generator network that produces novel molecular strings and a discriminator network aiming to distinguish between the *de novo* designs and existing molecules. Alternative DL approaches have been proposed for *de novo* design, for instance, molecular graph generation (Li et al., 2018b; Zhou et al., 2019) or fragment-based assembly (Jin et al., 2023c), but they have not been shown to outperform CLMs (Brown et al., 2019; Polykovskiy et al., 2020; Flam-Shepherd et al., 2022). Looking at the current progress and capabilities of powerful LLMs like ChatGPT and Bard, these CLMs can be used in various ways to assist drug discovery scientists, as mentioned below (Xue et al., 2023).

• *Screening large datasets of molecules*: LLMs may be used to sift through data sets to find compounds with required features. To achieve this goal, molecular descriptors, numerical representations of a molecule’s chemical structure, may be generated using LLMs. Descriptors may be used to group molecules with similar characteristics or to pick out compounds that fulfil certain requirements, such as binding to a specific protein.
• *Generating new drug candidates*: LLMs may be employed to generate potential novel molecules. After training, LLMs may be used to produce synthetic molecules resembling those in the training dataset.
• *Interpreting research data*: LLMs may be used to both understand patterns in experimental data from drug discovery research and create hypotheses about the underlying mechanisms of action. This strategy holds great promise for assessing the efficacy and safety of drug candidates.
• *Communicating with stakeholders*: Reports, presentations, and other documents developed with the help of LLMs may be shared with relevant parties such as scientists, doctors, and regulators. These models may help make decisions and answer questions from interested parties.
• *Repurposing existing drugs*: LLMs may be used to locate existing therapies that might be repurposed for new uses by analyzing the chemical structure of pharmaceuticals and finding potential new targets for commercially hit drugs.
• *Interpreting clinical trial data*: Using data from clinical trials, LLMs may be used to make hypotheses about the underlying mechanisms of action, assess the efficacy of existing drugs, and identify potential new therapeutic targets.

The large language model developed by the MIT/Tufts team (ConPLex) can match target proteins with potential drug molecules without performing the computationally intensive step of calculating the molecules’ structures. ConPLex can leverage the advances in pretrained protein language models (“PLex”) and use a protein-anchored contrastive coembedding (“Con”) to outperform state-of-the-art approaches. One recent review summarizes advances in AI-powered LLMs and their potential to aid drug discovery and development (Liu et al., 2021). Another review highlights opportunities for AI-powered LLMs in target identification, clinical design, regulatory decision-making, and pharmacovigilance (Chakraborty et al., 2023). To conclude, transformer, LLMs, GPT, BERT, and diffusion models are all interrelated in the context of generative AI. They are all based on the transformer architecture, a neural network architecture well suited for NLP tasks. The following Table 1 compares these GAI tools:

**TABLE 1:** Different types of GAI models.

Model	Description	Example applications
Transformer	A type of neural network developed by Google	Machine translation, text generation, question answering
LLM	Type of neural network that is trained on a massive dataset of text	Generation and translation of text, question answering, analysis of sentiment, named entity recognition
GPT	A LLM developed by OpenAI.	Capable of generating and translating languages, writing various unique content, and providing insightful answers to users’ prompts
BERT	A LLM developed by Google AI.	Particularly good at understanding the situation of words in a sentence. It can be used for sentiment analysis, named entity recognition, and question answering
Diffusion model	Type of LLM that is trained on a dataset of images	It can generate new images similar to those in the dataset.
Flow-based model	Type of generative model that explicitly models a probability distribution by leveraging normalizing flow	Image generation, density estimation, anomaly detection

Frequent GAI, research in non-pharma domains, rapid adoptions of this in the pharma domain, and ongoing findings indicate that various shades of basic and advanced autoencoders and transformers will keep coming for better results emanating from GAI.

## 4 Data requirement for GAI in drug discovery

To succeed, the design of a new drug must account for a wide range of chemistry and biology parameters, including the drug’s on-target potency, specificity with respect to off-targets, physical qualities, and more. The standard approach of scientists sifting through a huge chemical space to find promising candidate compounds and then proving their worth experimentally is inefficient. The widespread use of GAI models is attributable, to a great extent, to their efficiency and accuracy in generating novel bioactive and synthesizable compounds. The downside is that GAI models are famously data-hungry, and drug discovery databases are infamously tiny (e.g., in the range of 10^1^ to 10^4^ known biologically active molecules). Transfer learning has been widely used for chemical space exploration to make the most of limited data sets. It is a two-step process with the goal of applying skills learned while completing one activity to another. First, during pre-training, GAI models are typically trained (for example, for predicting the next character in a molecular string) on a massive dataset comprising 10^5^ to 10^6^ molecules. The second phase involves refining this pre-trained generic GAI model using a select group of molecules with the necessary characteristics (such as bioactivity towards a specific pharmacological target). In low training data regimes, GAI models have shown potential for various applications, including generating bioactive compounds inspired by natural products and simultaneously learning many attributes. Multi-factor interaction is required for GAI to explore the chemical space successfully. Target molecule complexity affects the minimal number of molecules needed to train a reliable model. More complex and heterogeneous molecules need more training data. The breadth of the chemical space explored is proportional to the structural variety of the training molecules, for instance, in terms of molecular frameworks and structural assortment. Despite evidence that fine-tuning with 10^1^–10^2^ molecules may result in empirically proven biologically active designs, doing so may need more stringent *post hoc* ranking processes to evaluate just high-quality structures exclusively. Augmenting SMILES can improve the CLM performance, although this benefit decreases when the augmentation factor is increased (e.g., after a 10 to 20-fold augmentation, as shown in. Small training sets of molecules (not more than 10,000) benefit the most from SMILES augmentation, whereas its impact does not change massively for bigger sets of data of structurally complicated molecules (more than 500,000), perhaps with the danger of over-enumeration and decline in quality. Though hyperparameter tweaking may have minimal influence on the CLMs’ performance, the number of epochs required for refinement alters the semantical quality of the output and is dependent on the size and variety of the dataset (Moret et al., 2023).

Active learning, multitask learning, one-shot learning, federated learning (a sort of ML in which the model is trained by utilizing separate private data held in multiple places without sacrificing data privacy), etc., are many other methods (other than transfer learning and data augmentation) that may be used to make the most of the insufficient data. The originality and reliability of the resulting molecule from GAI are affected by several variables, including the nature of the data and the GAI model used. A few recent, well-drafted review articles might be helpful to readers in this respect (Goldman et al., 2022). Evaluating GAI models and the quality of their output is often more difficult and time-consuming than AI-based predictive models. This is because GAI tools sometimes need users to prioritize competing goals simultaneously (such as increasing the resemblance to known bioactive compounds while simultaneously increasing structural uniqueness). Accordingly, doing a thorough case-by-case analysis of the structures is recommended by drawing on both domain experience and supplementary computational methods (such as pharmacophore models and molecular dynamics). One obstacle to a comprehensive assessment of GAI tools is the time and money needed for chemical synthesis. Rapidly navigating the chemical space using GAI models will be possible in the future with the help of self-driving laboratories (Schneider, 2018).

## 5 GAI in drug discovery: recent advances

Though at relevant places, we have discussed applications of GAI in molecular property prediction and *de novo* drug design, in this section, we will see a few recent applications of GAI in drug discovery. GAI models with a track record of effectiveness in other areas are finding growing use in the study of molecular generation. One recent work, probably the first to build on the success of ChatGPT, used the autoregressive model to create a tool called DrugGPT for ligand creation, emphasizing protein-ligand discovery and chemical space exploration (Li et al., 2023). To find new compounds that can associate with specific proteins, researchers in this work used the DrugGPT model to learn from a large quantity of protein-ligand binding data. The GPT-2 model was trained and optimized to meet the needs of the pharmaceutical industry. To accommodate the unique properties of proteins and ligands, they discarded the original tokenizer in favor of a BPE-based redesign and started training the GPT-2 model from scratch. DrugGPT can better record and comprehend drug compounds’ chemical rules and structural details with this update. It also improves its ability to interpret data on the interactions between proteins and ligands, which might lead to the development of novel therapeutics. To prevent the training process from becoming unstable due to the Mode Collapse issue of GANs, DrugGPT uses the back-propagation approach during model training. In addition, DrugGPT’s design philosophy gives it robust generalization capabilities, allowing it to learn and adapt to new tasks. GAI may be a new catchword lately, but Insilico Medicine, a drug development company, has used it for years. Early investment in DL is paying off for the firm, as a drug candidate found using its AI platform is moving forward into Phase 2 clinical trials for treating idiopathic pulmonary fibrosis. This rare respiratory condition causes a gradual deterioration in lung function. Insilico employed GAI throughout the preclinical drug development process to achieve this breakthrough, from identifying a new target to generating novel drug candidates to assessing the binding affinity of these candidates to the target to predicting the success of clinical trials. Using standard procedures would have required spending over $400 million and taking up to 6 years. Contrarily, Insilico could do these tasks using GAI in a third of the time and for a 10th of the expense, completing the first phase of clinical trials in little over two and a half years. In yet another study, the benefits of quantum GANs in generative chemistry were investigated. The authors presented a quantum-classical GAN hybrid for finding small molecules. The model’s quantum part is a variational quantum circuit (VQC), a quantum computer software suitable for molecular computations. The physicochemical features of the generated molecules and the model’s performance in a goal-directed benchmark were only two ways the authors demonstrated that the quantum GAN is superior to its classical counterpart. Based on their findings, the scientists think quantum GANs might be helpful in the field of drug development. In addition to being more effective than traditional GANs, the molecules they generate have superior characteristics. Nonetheless, the scientists acknowledge several remaining hurdles to overcome before quantum GANs are routinely employed in drug development. The scarcity of and the need for more sophisticated VQCs are contributing factors. Overall, the findings of this article are intriguing and show promise for using quantum GANs in the pharmaceutical industry. However, whether or not this promise may be realized requires more study (Mao et al., 2023).

Another study suggested a GAN based on a transformer for developing antiviral drugs. Researchers introduced a unique data-driven self-supervised pre-training generative model named “TransAntivirus” to perform select-and-replace edits on organic compounds and transform them into the necessary attributes for generating antiviral candidate analogues. The scientists proposed that molecular structures might be encoded in a more informative fashion by using transformers. They demonstrated that the transformer-based GAN is superior to previous GAN-based models in combating viruses. As a result of using this paradigm, it is possible to produce molecules with desirable characteristics like high binding affinity to the target protein and low toxicity. The model was also deemed more original, credible, distinctive, and varied than its baseline counterparts. As this is simply a proof-of-concept study, further investigation is required to evaluate whether transformer-based GANs can be utilized to identify novel medications in a realistic scenario. In addition, the scientists here only examined a tiny sample of compounds. It may be even more effective if the model were trained on a more extensive data set (Yu et al., 2023).

In another study, researchers detailed how they used generative models and structure-based drug design (SBDD) to find the macrocyclic CDK2 inhibitor QR-6401 more quickly. The scientists employed a generative model called FBVAE to develop a unique and proprietary lead CDK2 chemical 10 from previously reported CDK inhibitors. After using SBDD to improve compound 10 further, researchers isolated the macrocyclic inhibitor QR-6401. An IC50 of 0.46 nM was observed for QR-6401, indicating that it is highly active and selective against CDK2. The anti-cancer activity and oral bioavailability in an OVCAR3 ovarian cancer xenograft model were similarly promising. By combining generative models with SBDD, the scientists found that they could speed up finding novel medications. With QR-6401 as a proof-of-concept, generative models and SBDD are expected to be employed to find other effective drugs (Ren et al., 2023).

AlphaFold was recently employed to hasten the identification of a new CDK20 inhibitor. Before determining the exact fold of CDK20, the authors utilized AlphaFold to make educated guesses. The team then utilized this model to create a library of synthetic chemicals with binding predictions for CDK20. After synthesis and *in vitro* testing, it was shown that one of these compounds, ISM042-2-048, inhibits CDK20 activity in a particular and effective manner. AlphaFold, the scientists believe, may hasten the identification of novel medications by offering high-quality protein structures for use in generating computational compounds. Compared to conventional drug discovery strategies, their strategy only took 30 days to uncover a new CDK20 inhibitor. AlphaFold predictions may be used to create virtual molecules, which can then be utilized to discover new therapeutic targets. AlphaFold’s precise protein structures aid in discovering new therapeutic targets and quickening the pace at which new medications are brought to market (Drugdiscoveryonline, 2022).

A comprehensive review by Bai et al. delves into the application of DL and molecular docking in the field of *de novo* drug design. This method entails training a DL model to produce a molecular SMILES library. The generated molecular structures are then transformed into 3D ligands for docking with receptor targets. Binding affinity prediction for *de novo* drugs is conducted through virtual screening using DL models. Several tools, including SyntaLinker, Ligdream, GCPN, MolDQN, and others, utilize different DL techniques for molecular generation and optimization. Various models, including DeepDTA, Pafnucy, OnionNet, AttentionDTA, and GraphDTA, use deep CNNs and attention mechanisms to achieve precise binding affinity predictions between drugs and targets (Bai et al., 2022).

Below Table 2 shows some of the under investigation (clinical trial stage) molecules that emanated from AI:

**TABLE 2:** Some DL-derived, under investigation (under clinical trials) drug candidates.

Organization (s) involved*	Name of under-investigation compound (code name of clinical-stage assets)	Detail	Refs
Exscientia and Sumitomo Dainippon Pharma (which has expertise in GPCR drug discovery) [Centaur Chemist]	DSP-1181 (in clinical trial phase I)	A potent full serotonin 5-HT1A receptor agonist with a lengthy half-life against obsessive-compulsive disorder	Mullard, (2017); Burki (2020)
	DSP-0038 (in clinical trial phase I)	A dual-targeted agonist/antagonist for the 5-HT1a and 5-HT2a receptors against Alzheimer’s psychosis	Shimizu et al. (2022)
Exscientia and Evotec [Centaur Chemist]	EXS-21546 (in clinical trial phase I)	An adenosine A2a receptor antagonist for immuno-oncology therapy for several tumor types	Shimizu et al. (2022)
Exscientia and one co-owner	GTAEXS617 (in clinical trial phase I)	A selective and highly potent inhibitor of CDK7, being investigated against transcriptionally addicted cancers	Besnard et al. (2022), Businesswire, (2023b)
Exscientia and Bristol Myers Squibb	EXS4318EXS4318 (in clinical trial phase I)	Against Inflammatory Diseases	Businesswire, (2023b); Businesswire, (2023c)
Exscientia, under a collaboration with Sumitomo Pharma, referred to as design as a service or DaaS	DSP-2342 Phase I clinical trial will commence soon	A bispecific small molecule dual 5-HT2A and 5-HT7 antagonist with broad potential in psychiatric disease	Exscientia (2023)
Recursion [Recursion OS]	REC-2282 (Phase 2/3 Trial)	A possibly first-in-disease, orally active, central nervous system penetrant small molecule histone deacetylase (HDAC) inhibitor to treat progressive neurofibromatosis type 2 (NF2)-mutated meningiomas	Jayatunga et al. (2022)
	REC-4881	An orally effective, non-ATP-competitive allosteric small molecule against MEK1 and MEK2	Jayatunga et al. (2022)
Insilico Medicine 3 [Pharma.AI platform (PandaOmics3.0 for novel target discovery, Chemistry42 2.0 for molecule generation and optimization with ADMET prediction, InClinico 1.0 for clinical trial prediction)]	ISM001-055 and INS018-055 (in clinical trial phase I and II, respectively)	Compounds against idiopathic pulmonary fibrosis	Kirkpatrick (2022), Nagra et al. (2023)
	ISM3091 (in clinical trial phase I)	A ubiquitin-specific protease 1 (USP1) inhibitor that can potentially improve the result of cancer therapy by decreasing survivin levels and increasing DR5 through miR-216a-5p	BB (2023)
	ISM8207 (Phase I) (with Fosun Pharma)	A potentially first-in-class small molecule inhibitor of QPCTL for the treatment of advanced malignant tumors	Eurekalert (2023)
Verge Genomics 4-5 [CONVERGE]	VRG50635 (in clinical trial phase I) The company only took 4 years to bring this compound from research to the clinical phase	As mentioned in the text	Businesswire (2022)
BenevolentAI [Benevolent Platform]	BEN-2293 (in clinical trial phase I)	For atopic dermatitis	Richardson et al. (2020), Bess et al. (2022), Jayatunga et al. (2022)
BenevolentAI [Benevolent Platform] and Sheffield Institute for Translational Neuroscience (SITraN) at the University of Sheffield	BEN-34712 (company progresses this molecule for amyotrophic lateral sclerosis	An oral, potent, and selective brain penetrant RARɑ𝛃 (retinoic acid receptor alpha beta) biased agonist	Benevolent (2023)
Relay Therapeutics [Dynamo Platform]	RLY-1971/RG-6433 (in clinical trial phase I)	For solid tumors	Jayatunga et al. (2022)
Relay Therapeutics [Dynamo Platform]	FGFR2 (in clinical trial phase I)	For FGFR2-driven cancers	
Nimbus Therapeutics	NDI-010976/GS-0976 (in clinical trial Phase II)	For nonalcoholic steatohepatitis (NASH)	
Pharos iBio [Chemiverse]	PHI-101 (in clinical trial phase I)	For acute myelogenous leukemia, platinum-resistant refractory ovarian cancer, and other cancers	

As far as DSP-1181 is concerned, as per the information on the internet, further research (clinical studies) on it in Japan has been stopped since the Phase I study did not meet the encouraging output (Schneider, 2018; Company Bap, 2022). Similarly, the clinical trials of BEN-2293 (a clinical-level asset from BenevolentAI) led to inclusive efficacy against atopic dermatitis, as per multiple websites. According to information on the company website, the company is currently evaluating the results of this trial for further processing of this drug candidate (Adcreviews, 2023; Benevolenet, 2023; BenevolentAI, 2023; Businesswire, 2023a; Clinicaltrialsarena, 2023).

## 6 GAI in drug discovery: challenges and opportunities

AI has recently taken the drug development industry by storm, allowing researchers to generate entirely novel compounds from scratch. The crucial driving force (Bickerton et al., 2012) of the international pharmaceutical business remains small molecules; therefore, GAI technologies are here to stay and reshape the current ways to discover or design drug molecules. There is growing evidence that GAI may be used to investigate hitherto undiscovered areas of chemistry. This is mainly due to the simplicity with which molecular strings (in the case of CLMs) can be generated and used and the breadth of tasks to which they can be put. We anticipate that future developments in LLMs/NLP algorithms and the addition of medicinal chemistry skills will further drive GAI’s capabilities in drug discovery. Despite GAI’s potential, many obstacles must be overcome before it can be extensively used in drug development. Optimizing toy yardsticks (such as molecular weight, octanol-water partitioning coefficient, or the quantitative estimation of drug-likeness (Renz et al., 2019), is a common way to assess the efficacy of GAI models. These goals capture the capability to generate compounds that meet set criteria, but they do not do justice to the complexity of actual drug development (and their exact behaviour/fate in biological system) and may result in simplistic answers (Merk et al., 2018b; Moret et al., 2021). Although they do not entirely address the quality of the generated compounds, existing *de novo* design benchmarks (such as GuacaMol (Brown et al., 2019) and MOSES (Polykovskiy et al., 2020), provide a way to ensure comparison across techniques developed separately.

Due to the computational difficulties inherent in assessing the quality of *de novo* designs, experimental validation represents the gold standard. Thus far, only a handful of potential GAI applications have been published (Li et al., 2020b; Grisoni et al., 2021; Moret et al., 2021). Implementing GAI into practical use will need cooperation amongst DL experts, cheminformaticists, and medicinal chemists. Although automated synthesis platforms may restrict the chemical space available for synthesis, they may be a viable alternative to speed up *de novo* design driven by GAI (van Tilborg et al., 2022).

Though BERT and GPT models have been used to comprehend molecular structures in the case of chemical compounds, their effect on drug development has not been particularly noteworthy thus far. Predicting DTIs computationally is another hurdle. The lack of labelled data is a significant challenge in training models working with peptides or proteins rather than small compounds. However, if LLMs can handle peptide and protein sequences as text, they may be able to make a difference in this field. In addition, with the latest advancements in DL, we can now predict which medication combinations will be most successful against complicated illnesses by gaining insight into the interaction between small compounds and genetic alterations. This demonstrates the promise of building GAI models that integrate data from many linked domains, such as chemical structures and gene expression. Large CLMs may be developed similarly to LLMs by learning from existing examples of such models.

The importance of conditional generation algorithms is predicted to grow in the coming years. These techniques may enable the generation of molecules designed to meet specific requirements, potentially overcoming the limits of current scoring systems (for instance, because of non-additivity, activity cliffs (Kwapien et al., 2022; Özçelik et al., 2022). A promising structure-based design may address *de novo* design for as-yet-undiscovered macromolecular targets (Volkov et al., 2022), by producing molecules that match specific binding sites’ electrostatic and shape properties. Potential shortcomings and bias in available protein-ligand affinity databases may account for the lacklustre practical implementation of structure-based *de novo* design (Graff et al., 2021). There is untapped potential in fields like polypharmacology and selectivity that might benefit significantly from fine-grained control over various aspects of *de novo* designs. Exorbitant expense (of GAI setup) and biased GAI models are further obstacles. If drug candidates are generated using biased models, they may be unsafe, ineffective, or reflect the biases of the experts. Cloud computing is one option to overcome the problem of high processing costs. Thanks to cloud computing, researchers no longer need to invest heavily in expensive computer gear to access extensive computing resources. The time and money required to train GAI models (and address the issue of inadequate data) may be cut drastically. As previously said, another option is to use transfer, active, and federated learning. Alternatively, one might try several data augmentation methods. As a result, GAI models may be less susceptible to bias due to data noise. Debiasing methods are another option since they may be used to either discover and eliminate biased features from the data or to alter the model’s weights to lessen the bias (Taly et al., 2019; Abdel-Aty and Gould, 2022).

Future applications of GAI models are anticipated to be bolstered by few-shot learning methodologies paired with large-scale pre-trained CLMs (Gao and Coley, 2020). The practical usefulness of CLMs in drug development is also likely to grow as their capacity to suggest synthesizable compounds is enhanced (Krenn et al., 2022). Expanding chemical languages to include increasingly complicated molecular entities, such as proteins and peptides with non-natural amino acids, also holds considerable promise for expanding the use of GAI tools in chemistry. Recent discussions (Bender and Cortés-Ciriano, 2021), have gone further on how SELFIES may be further developed to tackle the problems with existing molecular string representations, and these ideas could inform new iterations of SMILES and DeepSMILES as well. The function of GAI models in the drug development process is anticipated to grow in importance. GAI will improve time and cost efficiency by accelerating our ability to explore undiscovered parts of the chemical space and to create and test fascinating new scientific ideas for drug discovery. Experts in AI, chemistry, and biology will be able to work together in the future to create cutting-edge algorithms infused with scientific information and to acquire AI-powered novel insights into human biology. In their two reviews, Bender et al. (Gao and Coley, 2020; Bender and Cortés-Ciriano, 2021), explained what has been achieved and what lacunas are hindering major fruits in AI-driven drug discovery. Readers are referred to these two articles for an in-depth analysis of why other domains are getting more benefits from AI *versus* biological and chemical domains, primarily the drug discovery domain (Zhang et al., 2023).

## 7 Summary and future directions

The development of GAI has had a profound impact on finding novel drugs. This article examines where GAI models are and how they may help researchers find new drugs. The molecular generators based on simple CNNs, GNNs, and RNNs may be somewhat efficient. All VAE, GAN, and related methods use a variational inference strategy. RL differs from variational inference because it allows real-time process tuning to produce the unique molecules of interest. There is great potential for these GAI methods, together with highly effective and modern transformers and LLMs, to profoundly alter the area of drug development. However, GAI models need vast and varied datasets of compounds to learn to produce new molecules with desirable features, even though current development and quick advancements in the algorithm imply that the future of GAI in drug discovery is quite promising. However, getting access to such databases may be time-consuming and costly. Although many techniques exist for dealing with insufficient data, new methods need to be developed so that GAI may be used to its full potential, just as in other fields. Research into various AI applications (such as autonomous vehicles, computer vision, and NLP) may be used for AI-driven drug development since the underlying algorithms are universal and need domain-specific adjustments to meet the particular requirements of chemical compounds and biological macromolecules. More and more scientists, enabled by today’s technology, are beginning to appreciate parallel and distributed computing for its many benefits. Furthermore, GAI models need to be interpretable to be helpful for drug development. This necessitates that researchers know why and how the model creates certain chemicals. There must be a high degree of data robustness in GAI models. This is because the training data for these models is often imperfect and contains noise. Despite these limitations, GAI models are promising to revolutionize drug discovery. These models can potentially speed up and lower the overall cost of drug development by automating the generation of novel compounds. There is a good chance that GAI models will play a crucial part in developing novel drugs as technology advances and GAI models become more sophisticated and as the difficulties of data insufficiency, model interpretation, bias, and computational expense are overcome. We are certain that GAI will revolutionize the drug discovery process and that, when combined with more conventional methods, we will see remarkable advances in the generation of life-saving compounds.
